# Dispersal potential of a tidal river and colonization of a created tidal freshwater marsh

**DOI:** 10.1093/aobpla/pls050

**Published:** 2012-12-21

**Authors:** Mary Allessio Leck

**Affiliations:** Department of Biology, Rider University, 2083 Lawrenceville Road, Lawrenceville, NJ 08648, USA

## Abstract

**Background and aims:**

A created tidal freshwater wetland, with no extant seed bank, was monitored to evaluate the colonization potential of a tidal river, vegetation and seed bank changes, and also species persistence.

**Methodology:**

The 32.3-ha mitigation, adjacent to the Delaware River, was completed in 1994. Three sites, with differing completion dates, and three locations (channel edge, midpoint, upland edge) were sampled. From 1995 to 1999, surveys permitted a view of initial dynamics involving soil seed bank density or field vegetation cover, as well as species composition. Vegetation was monitored through 2011, and in 2011 a seed bank survey was also undertaken.

**Principal results:**

Between 1995 and 1999, 177 species emerged from soil samples, with a maximum of 32.3 ± 1.8 (SE)/sample. Eight species were added in 2011, indicating continuing dispersal and input. Comparing species richness across three sites for midpoint locations, 2011 totals and species/sample were lower (61–90 % and 53–72 %, respectively) than 1998 values. In vegetation plots during the first 5 years, 72 cover species were present. In 2011, there were 44; 14 were new and the majority (13) were woody and part of an overhanging edge canopy. Dispersal was primarily via water. Overall, the trend was towards lower seed bank densities, except for *Mikania scandens* and *Phragmites australis* which had higher densities, as well as cover values. Species richness also decreased. Species varied in persistence in the seed bank and vegetation, and among sites and with location. Initial site colonization differences were still apparent in 2011 seed bank samples. While present, *Lythrum salicaria* did not reduce species richness; *Phragmites*, in contrast, had substantial impact.

**Conclusions:**

The single most important change was an increase in *Phragmites* in the vegetation. Vegetation dynamics are now determined by this species, and the persistent seed bank and continuing dispersal input have little obvious future in vegetation development.

## Abstract (precis)

Over 17 years, species richness and seed bank densities, initially very high, decreased, and *Phragmites australis*, an invasive, came to dominate the seed bank and vegetation. Despite a persistent and varied seed bank, *Phragmites* will likely preclude contributions from the seed bank or newly dispersed propagules to vegetation development.

## Introduction

The Abbott Marshlands (also known as the Hamilton–Trenton–Bordentown Marsh) include the northernmost tidal freshwater wetland in the Delaware River estuary, where the tidal range may exceed 3 m. The wetland has been the subject of a number of ecological studies dating back to 1974 (Whigham and Simpson 1975) that addressed productivity, sedimentation and water quality, as well as seed banks (for reviews see Leck and Crain 2009; Leck *et al*. 2009; Whigham 2009). Initial colonization in a created wetland was also documented and this report is a follow-up of those studies (Leck 2003; Leck and Leck 2005).

### Created wetland—1995–99 overview

**Fig. 1:**
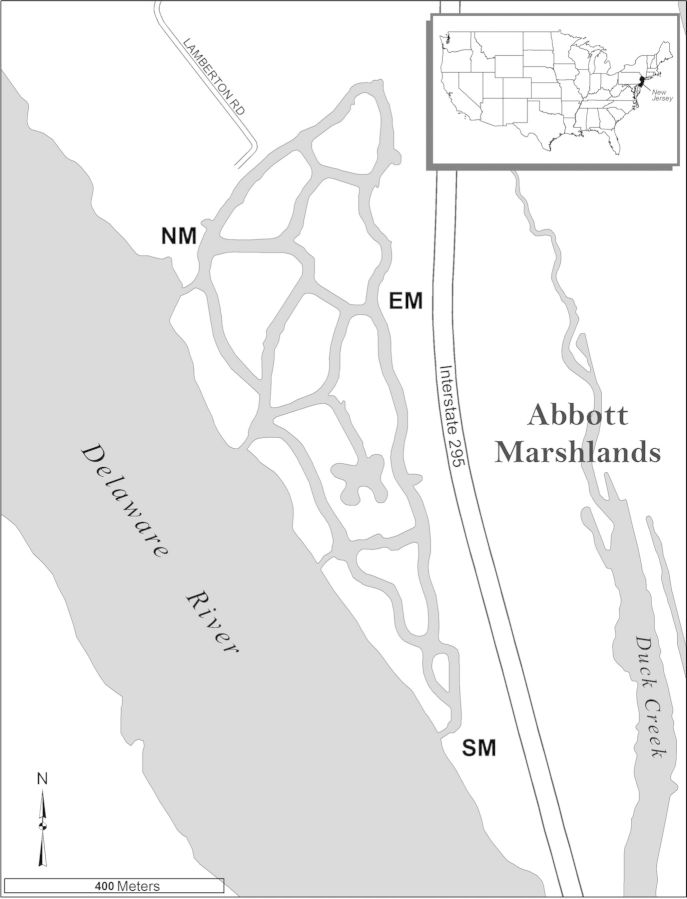
**The Abbott Marshlands created wetland, Mercer County, New Jersey, USA**. NM, north marsh; EM, east marsh; SM, south marsh. The created wetland on Duck Island lies at 74°43′30″ longitude, 40°10′46″ latitude and is 3.5 km south of Trenton, New Jersey, 107 km west of New York City, and 47 km north of Philadelphia, PA. The site, which covers 43 ha and is 1.5 km long, was constructed between 1993 and 1994. It is the largest New Jersey Department of Transportation mitigation project in the state and is located in an urbanized and industrial landscape. It is ∼2 km north of the mouth of Crosswicks Creek which drains most of the Abbott Marshlands. There are two 1-m pipes beneath the highway that connect the created wetland with Duck Creek. To the east of Duck Creek lie the D&R Canal and a railroad track (not shown) that further isolate the created wetland from the main part of the Abbott Marshlands.

The created wetland was constructed in stages, beginning in January 1993 and extending to November 1994. The completed project had eight islands, edge marshes and ∼4 km of channels (Fig. 1). The study objectives (Leck 2003) were to document seed bank and vegetation development. At the time the study was initiated during early May 1995 in three edge marshes, vegetation, dominated by *Juncus effusus*, was well developed along the east marsh [see Additional Information]. The north marsh, in contrast, had little vegetation except for a few *Salix* and *Populus* saplings growing along the channel edge that did not persist, and *Typha latifolia* in an ∼5-m-wide depression; a number of wetland species were present in 1994 along the upland edge, including *Lythrum salicaria*, *Sagittaria latifolia* and *Symphyotrichum puniceum*. There was no vegetation in the south marsh, the last area to have been completed (November 1994). However, by mid-August 1995 at each site, vegetation cover was complete and diverse, and *Lythrum* was in flower (M. A. Leck, pers. observ.).

Over the first 5 years the following observations were made: (i) the range of construction times, between November 1993 and November 1994, for the three sites provided different establishment and gap opportunities that resulted in varied and distinctive seed banks and vegetation; (ii) the quality of germination niches changed as vegetation developed and sediment deposition altered conditions; (iii) spatial and temporal variability in the seed bank and vegetation among sites, locations and years were related to deposition of seed-containing wrack, species tolerances to tidal flooding and *in situ* seed production; (iv) the relative importance of regional versus local dispersal changed with *in situ* seed production, becoming more important and resulting in high densities (>350 000 m^−2^) in some locations; (v) seeds of most colonizing species were small and persistent; (vi) seed dispersal was primarily by water; (vii) seed bank and vegetation compositions became increasingly similar with time; and (viii) persistence in the seed bank and vegetation varied with species (Leck 2003).

Both seed densities and species diversity of the created wetland were substantially higher than those of a nearby reference marsh site (Leck and Leck 2005), similar to results from a freshwater wetland along the Anacostia River in Washington, DC (Baldwin 2004). A Napa River (California) brackish tidal marsh study found the opposite pattern, with reference sites having higher diversity than a restored one, indicating limited dispersal there (Diggory and Parker 2010).

Owing to the proximity of the created wetland to the Delaware River, the high seed bank and vegetation diversity initially observed and the potential of the tidal river for continuing dispersal, it was expected that diversity would continue to be high especially in the seed bank. Accordingly, the main objectives of this study were to compare data from early seed bank and vegetation studies with those obtained over 17 years at the same sites, and to explore the impact of continuing seed dispersal into the created wetland on both the seed bank and vegetation development. Also of interest were: whether the extraordinarily high species richness present in the early study was maintained and if input of species continued despite the presence of established vegetation; how the seed bank and vegetation developed over time; how persistence of species varied in the seed bank and vegetation; and how invasive species contributed to species dynamics. This study appears to be the first to document seed bank and vegetation changes in a created tidal freshwater wetland over 17 years.

## Methods

### Sites and locations

The three edge sites selected—north (NM), east (EM) and south (SM) marshes—were accessible by foot (Leck 2003) (Fig. 1). At each site, three transects perpendicular to the tidal channel were established; transects were 30–35 m long, except for one in the NM that was 15 m, and were ≥10 m apart. Sampling locations were 1 m from the channel edge (ch), near the midpoint (mi) and 1 m from the upland edge (up). At each location, three replicate points were designated, adjacent to and 1 m at either side of the transect marker. For seed bank samples, distances varied to avoid places already sampled. For vegetation, typically, there were nine samples for each location at a site. For seed bank data reported here, typically *n* = 6, except for June 1999 when *n* = 3. In later years, samples were occasionally lost or a plot missed due to difficulties in finding locations along transects overgrown with *Phragmites australis* and *Mikania scandens*.

### Sampling

#### Seed bank

Samples, 10 cm × 10 cm × 3 cm deep, were collected on 6 May 1995, 15–20 April 1996, 27–28 March 1997, 16 March 1998, 18 March and 16 June 1999, and 18/19 March and 18 June 2011. In 1999 and June 2011, only midpoint locations were sampled. Within 24 h of collection, samples were spread out in 20 cm × 20 cm aluminium pans over a 2-cm layer of perlite, except in 1995 when sand (1 cm deep) was used; placed in a greenhouse with ambient photoperiod, ∼30 % solar illumination, and air temperature in the range ∼5–30 °C; and were watered regularly. Although they were usually at field capacity, the 2011 samples experienced some drying.

Seedlings that emerged from soil samples were used to estimate the size (m^−2^) and species composition of the seed bank. Samples were monitored weekly and seedlings were removed when they could be identified. However, during May and early June 1995–99 when seedlings were very dense, seedlings (<2 mm) were removed before they died. Representative unknown seedlings were transplanted to pots and maintained until they could be identified. In the case of small seedlings of *Lindernia dubia*, *Ludwigia palustris*, and *Lythrum*, which were difficult to distinguish, seedlings were designated as ‘dicots’, and an undisturbed patch was left to grow; once identified, the ratio between species was calculated and the number of each determined. Although samples were maintained for more than one growing season to detect any ungerminated species, only data up to December of the year collected are presented.

Persistence of seeds was determined by using samples collected in June after the transient seed bank component would have germinated but before the current year seed production dispersed, and 3–6 cm depth samples (Leck 2003). Depth samples, used to evaluate the incorporation of seeds of undetermined age into the soil profile, were collected in 1998 at one-third of the sampling points (*n* = 3) at each location for each site and in 2011 at SM midpoint locations (*n* = 6). It was difficult to avoid contamination of 3–6 cm samples due to the unconsolidated nature of the substrate and the flow of water into the hole. June samples were collected in 1999 and 2011.

#### Vegetation

From 1995 to 2011, the presence and per cent cover were determined for 14 years using 50 cm × 50 cm quadrats. Evaluation dates were: 16 August 1995; 15 August 1996; 22 August 1997; 21 August 1998; 28 August 2000; 1 September 2001; 26 August 2002 (NM, SM only); 6 September 2003; 22 August 2004; 22/23 August 2005; 23 August 2006; 2/3/5 September 2009; 24/29 August/6 September 2010; and 12/13/17 August 2011. In recent years, when midpoint location markers that had been overgrown and hidden by *Phragmites* and/or *Mikania* could not be located, plot locations were estimated.

Between May and September 1999, a clipping experiment was undertaken to determine the impact of *Lythrum* on biodiversity (Licsko 2000). Five replicate treatments plots, 2 m × 2 m, for each of five treatments were set up in the EM and SM. Treatments were: clipped 1× (May), 2× (May, July), 9× (biweekly), standing dead/litter (thatch) removed (May), and control. To estimate the per cent cover and species present, a 1-m circular plot was established within each treatment plot. The Spearman correlation coefficient was used to determine if the cover of *Lythrum* influenced the total number of species or number of cover species.

Seed bank and vegetation results are presented as total species, species richness (*x* ± SE of species/sample or /vegetation plot), estimated seed bank densities m^−2^ (*x* ± SE) and % vegetation cover (*x* ± SE/plot). Composition comparisons were made with the Sørenson coefficient (Brower *et al*. 1998).

Nomenclature follows USDA (2012). Non-native status was determined from Rhoads and Klein (1993), and rare plants, those rated as threatened or endangered for New Jersey, from Snyder (2010).

## Results

### Seed bank and vegetation diversity

Diversity was higher in the seed bank than in vegetation (Table 1). Overall 194 species appeared in both seed bank samples and field plots, representing more than 54 families. Families with the most species were Asteraceae, Cyperaceae, Poaceae and Polygonaceae. In 1998, there were nearly twice as many families in seed bank samples than were present in vegetation plots, compared with the nearly equal numbers in 2011.

**Table 1:** **Diversity summary for seed bank and vegetation, comparing 1995–99 with 2011.** (A) Total species richness (*x* ± SE), and non-native and rare species (globally or New Jersey state endangered or threatened; Snyder 2010). Also indicated are sites (NM, EM, SM; see Fig. 1), locations (ch, mi, up) and year. (B) Comparisons of seed bank and vegetation data for three time periods. Data are the numbers of plant families and of species for the four most abundant families. *n* = 6 for seed bank data and *n* = 9 for vegetation plots. Seed bank soil samples were 10 cm × 10 cm × 3 cm; vegetation cover plots were 50 cm × 50 cm. Overall 194 species appeared in 1995–99 and 2011 samples and plots.

A						
	1995–99	2011				
Seed bank						
Total species [5-year range]	177 [69–111]	70				
% Non-natives	13	17				
% Rare species	3 (4.5)^a^	(3.2)^a^				
Species richness range	3.3 ± 1.3–32.3 ± 1.8	6.2 ± 2.5–12 ± 1.1				
Site location year	SM mi 95/NM up 98	SM ch 11/SM up 11				
Vegetation						
Total species [5-year range]	92 [33–48]	44				
% Non-natives	12	10				
% Rare species	16	5.7				
Species richness range	1.8 ± 0.2–9.4 ± 0.86	1.8 ± 0.3–6 ± 0.7				
Site location year	EM ch 95/SM mi 96	EM ch 11/NM up 11				

B						
	Seed bank			Vegetation		
	1995–99	1998	2011	1995–99	1998	2011
Families—total	54	51	28	25	23	29
Species						
Asteraceae	29	20	11	8	8	4
Cyperaceae	33	16	6	16	6	5
Poaceae	23	11	7	5	4	4
Polygonaceae	13	8	4	10	4	2

^a^*Heteranthera* and *Mimulus* seedlings did not flower, but values in parentheses could include *H. multiflora* or *M. alatus* which are rare.

The maximum seed bank species richness (32.3 ± 1.8/sample), obtained in 1998, was more than 2× higher than for any other year. The lowest seed bank richness occurred in 1995, but in the vegetation similar low values occurred in the EM channel location in 1995 and 2011. In 2011, eight species were added to the seed bank inventory and five to the vegetation (Appendix 1). Of the new seed bank species, three occurred in channel edge samples and of these only *Echinochloa muricata* was a wetland species (FACW+).

Over time, the upland adjacent to the tidal marsh was colonized by woody species that contributed cover to the upland edge vegetation plots, but were not rooted in the plot. In 2011, 15 woody species, including vines (e.g. *Clematis terniflora*, *Lonicera japonica* and *Vitis* spp.), were recorded (Appendix 1). Of these, seven were also present in seed bank samples.

The Sørenson coefficients, used to compare the location composition of seed bank/vegetation or 1998/2011, were generally low (<50 %) (Table 2), while values comparing sites were high (>50 %; N/E = 57 %; E/S = 57 %; N/S = 71 %). The values comparing March/June seed bank compositions were also high (55–77 %). When woody species, not rooted in the vegetation plots, were not included (1998/2011), the similarity was low.

**Table 2:** **Sørenson coefficients (%) comparing species in March and June 2011 with 1998 for seed bank samples and vegetation cover plots.** Values are for three sites (NM, EM and SM, shown in Fig. 1), and three locations (ch, mi and up). If all sites and locations are included for 1998/2011 comparisons, the seed bank value is 47 % and that for the vegetation is 47 %.

Site–location	2011	2011	1998 : 2011
	Seed bank March : vegetation	Seed bank March : June	Vegetation
NM–ch	42		58
NM–mi	50	55	60
NM–up	38		49
NM–up, no woody species			49
			
EM–ch	33		38
EM–mi	22	56	24
EM–up	10		10
EM–up, no woody species			8
			
SM–ch	36		37
SM–mi	33	77	22
SM–up	13		19
SM–up, no woody species			12

Non-native species comprised 10–17 % of totals and proportions were equally distributed between seed bank and the vegetation during the initial stages of colonization (Table 1). Rare species ranged from 3 to 16 % and were, however, most numerous in the vegetation during the initial stages of colonization. A few found elsewhere in the created wetland, e.g. *Isoetes riparia* (G4, S3) and *Elatine americana* (G4, S2), were restricted in occurrence. In fact, a few *Isoetes* plants were observed only in 1994 in a small depression. Other species, namely *Heterantera multiflora* (G4, S3) and *Mimulus alatus* (G5, S3), were more abundant and frequent (M. A. Leck, pers. observ.).

### Changes

Comparisons among sites (NM, EM, SM) and among locations (channel edge, near the midpoint, and upland edge) will be presented as follows. Site comparisons will use midpoint location data for 1998 and 2011, and all locations for 2011. Location changes will focus on the SM.

#### Sites

Sites showed substantial reduction in the total seed bank density and diversity between 1998 and 2011, with values generally being >50 % less in 2011 (Table 3). Vegetation diversity was also less in 2011 with richness ranging from only 2.0 ± 0.2 to 3.2 ± 0.7 species/plot. The relatively high NM vegetation species totals in 2011, compared with EM and SM, were due to the lack of *Phragmites* in some NM locations.

**Table 3:** **Comparisons of seed banks and vegetation for 1998 and 2011 at NM, EM and SM midpoint locations (see Fig. 1).** Data are seed bank densities m^−2^ (*x* ± SE) and % cover (*x* ± SE) for selected species, species richness (*x* ± SE) and total species. For the seed bank data typically *n* = 6, and for the vegetation *n* = 9.

	Seed bank		Vegetation	
	1998	2011	1998	2011
NM				
*Gratiola neglecta*	17 317 ± 4222	17 ± 14	0	0
*Impatiens capensis*	200 ± 141	100 ± 67	3.3	1.1 ± 1.1
*Juncus acuminatus*	2883 ± 1076	467 ± 222	1.6 ± 1.2	0
*Juncus effusus*	1450 ± 729	17 ± 14*	0	0
*Lindernia dubia*	59 800 ± 14 985	450 ± 238	0	0
*Ludwigia palustris*	258 620 ± 34 081	1183 ± 391	4.1 ± 2	0
*Lythrum salicaria*	12 133 ± 3331	18 750 ± 554	27.7 ± 6.3	18.3 ± 6.4
*Mikania scandens*	17 ± 17	13 533 ± 1089	9.4 ± 5.7	41.1 ± 12.1
*Phragmites australis*	100 ± 37	3683 ± 1043	0	53.9 ± 14.5
*Pilea pumila*	3983 ± 1653	17 ± 14	20.6 ± 6.8	0
Total density	382 080 ± 38 649	42 067 ± 7353		
Species/sample	28.8 ± 3.0	11 ± 1.3	6.8 ± 0.6	3.2 ± 0.7
Total species	63	25	18	12
				
EM				
*Gratiola neglecta*	500 ± 171	50 ± 28	0	0
*Impatiens capensis*	50 ± 34	0	6.1 ± 4.4	0
*Juncus acuminatus*	233 ± 196	17 ± 14	0	0
*Juncus effusus*	10 283 ± 1977	15 400 ± 3078	1.1 ± 1.1	0
*Lindernia dubia*	817 ± 234	117 ± 49	0	0
*Ludwigia palustris*	97 083 ± 29 730	4117 ± 1235	0	0
*Lythrum salicaria*	25 800 ± 5507	21 417 ± 6317	28.9 ± 8.9	0
*Mikania scandens*	1300 ± 583	9367 ± 1290	14.1 ± 6.7	28.3 ± 12.7
*Phragmites australis*	0	10 383 + 2974	0	84.4 + 2.3
*Pilea pumila*	4416 ± 2109	300 ± 143	12.2 ± 4.4	0
Total density	165 370 + 32 408	63 383 + 4797		
Species/sample	29.2 ± 1.2	11.8 ± 1.3	7.8 ± 0.6	2 + 0.3
Total species	58	24	21	4
				
SM				
*Gratiola neglecta*	3967 ± 1618	50 ± 28	0	0
*Impatiens capensis*	217 ± 95	0	60.6 ± 9.0	0
*Juncus acuminatus*	8967 ± 7423	33 ± 17	0	0
*Juncus effusus*	233 ± 105	0	0	0
*Lindernia dubia*	58 116 ± 25 607	300 ± 123	0	0
*Ludwigia palustris*	174 730 ± 31 109	2517 ± 729	0	0
*Lythrum salicaria*	27 216 ± 7178	5617 ± 1662	15.4 ± 5.4	6.7 ± 5.4
*Mikania scandens*	133 ± 61	14 600 ± 3299	0	25.8 ± 7.6
*Phragmites australis*	0	5400 ± 1213	0	78.3 ± 5.2
*Pilea pumila*	3050 ± 742	17 ± 14	6.1 ± 2.5	0
Total density	305 460 ± 49 903	29 504 ± 4880		
Species/sample	28.7 ± 1.5	8 ± 0.4	5.0 ± 0.4	2.0 ± 0.2
Total species	66	14	14	4

While the density of most species decreased with time, *Phragmites* and *Mikania* increased in importance at all sites. These species also became important components of the vegetation. Several others (e.g. *Lythrum* and *J. effusus*) were absent from the vegetation although they were well represented in the seed bank in 2011. Species varied with site; for example, values for *Lythrum* in NM and EM were similar, but much reduced in the SM.

For 2011 across sites (Appendix 2), channel locations had the lowest seed bank densities and species richness. Total species were similar at midpoint and upland edge locations. Many species present in the seed bank were not present in the vegetation, and richness was much lower and fewer species contributed to the vegetation. In EM and SM locations, which tended to be more alike than either site compared with the NM site, only 2–3 species contributed most of the cover. *Polygonum punctatum* and *Nuphar lutea* were important along the channel, and *Phragmites* in midpoint plots. *Phragmites* was the only important herbaceous species in upland edge plots, but had reduced cover (compared with the midpoint locations). Seed bank density and species richness were lower than in earlier years (see Table 3, Appendix 2; Leck 2003), but total species remained fairly high.

#### Locations

**Fig. 2:**
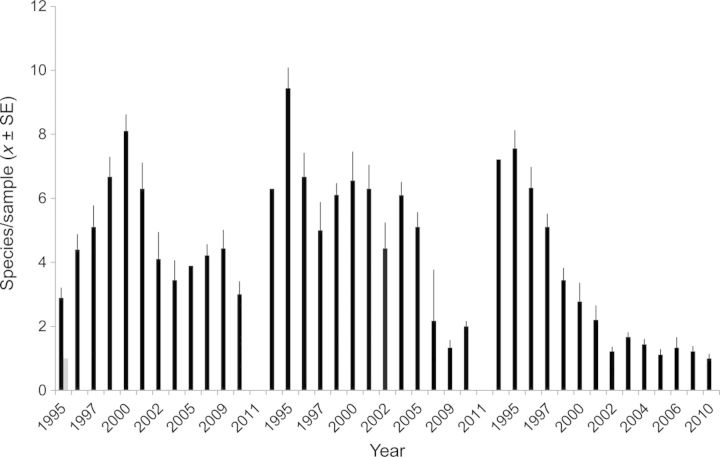
**Vegetation species richness in SM locations for 1995–2011.** Locations: left, channel edge; centre, midpoint; right, upland edge. Data are for 14 of 17 years. Values are *x* ± SE/plot; typically *n* = 9. Plots were 50 cm × 50 cm.

Species richness of vegetation at all three SM locations (Fig. 2) was initially high and then declined, especially in midpoint and upland edge plots. However, patterns differed among locations: the channel edge showed an initial increase and then was fairly stable; midpoint plots were fairly constant until the last three sampling dates; and along the upland edge species richness declined after the first 2 years. Location changes in total species showed similar site patterns (data not shown). In recent years, species richness was lowest at midpoint locations, and increased at upland edge locations.

**Fig. 3:**
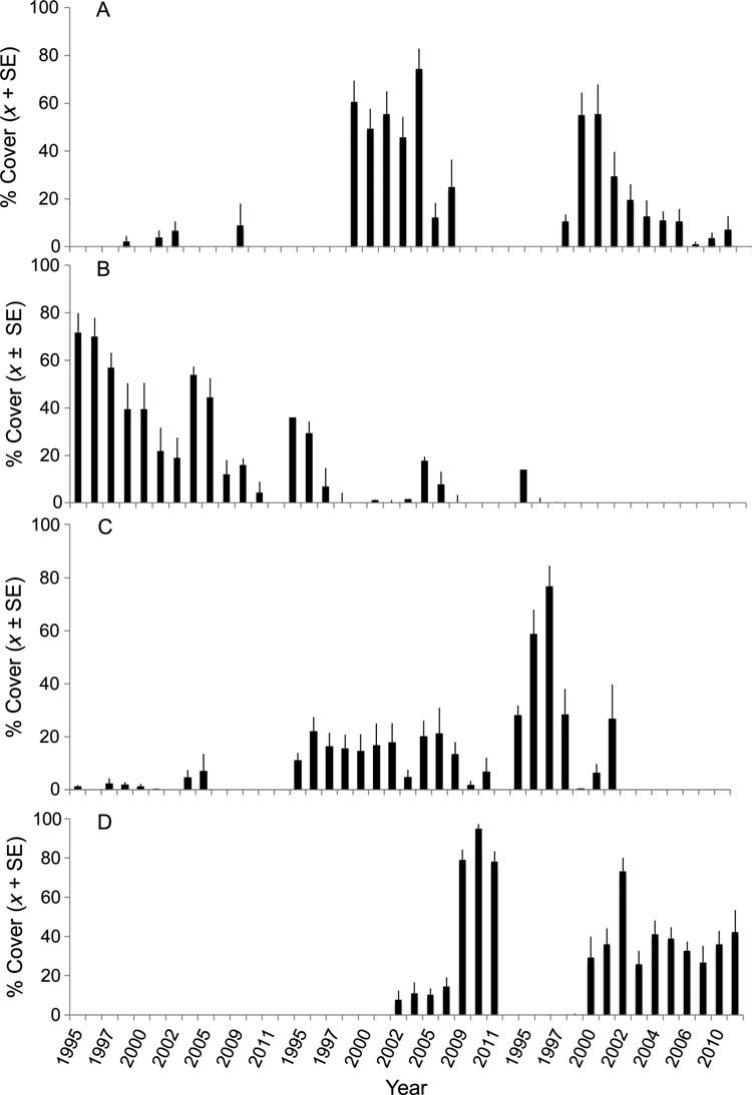
**Cover (%) in SM locations from 1995–2011 for four species: annuals**, **(A) *Impatiens capensis*, (B) *Polygonum punctatum*, and perennials, (C) *Lythrum salicaria* and (D) *Phragmites australis***. Locations: left, channel edge; centre, midpoint; right, upland edge. Data are for 14 of 17 years. Cover values are *x* ± SE/plot; typically *n* = 9. Plots were 50 cm × 50 cm.

Individual species waxed and waned in unique ways. *Impatiens*, for example, did not immediately colonize the SM site although seedlings were noted elsewhere in the created wetland in 1994 (M. A. Leck, pers. observ.) and was not a continuing presence along the channel edge (Fig. 3A). However, by the third year, *Impatiens* was well established at midpoint and upland edge locations, and persisted for several years, but eventually was lost from the vegetation.

*Polygonum punctatum* was an important initial colonizer of the vegetation along the channel edge beginning in 1995 and, although it was found in 1995 and 1996 at higher elevations, it did not persist there (Fig. 3B). It should be noted that identification of *Polygonum* species that are not flowering can be problematic, and during the first 2–3 years several species were present. The decline of *P. punctatum* was probably related to slumping and erosion of the channel edge, and expansion of *Nuphar* from the stream channel. Cover values of *Nuphar* in the SM channel plots increased from 0 % in 2004, to 20 ± 10.1 % in 2005, 16.7 ± 4.8 % in 2006, 35.7 ± 9.6 % in 2009, and 27.8 ± 8.9 % in 2010 (*n* = 9).

### Persistence

Many species germinating from June samples have persistent seeds (Table 4). *Lythrum*, *Mikania* and *Phragmites* were the most numerous in June 2011 samples from all sites. In 1999, seed bank dominants, besides *Lythrum*, included *Ludwigia* and, depending on the site, *Lindernia*, *J. effusus* or *Juncus acuminatus*. Site seed banks varied, and *J. effusus*, while not present in any vegetation plot after 2001, was present in 2011 in sizeable numbers but only in EM samples. In 2011, there were 62 total species in March samples compared with 24 in June.

**Table 4:** **Comparisons of seed banks for June 1999 and June 2011 at NM, EM and SM midpoint locations (see Fig. 1).** Data are density m^−2^ (*x* ± SE), species richness (*x* ± SE/sample) and total species.

	1999	2011
NM		
*Juncus acuminatus*	3930 ± 2740	50 ± 28
*Lindernia dubia*	13 780 ± 4750	233 ± 128
*Ludwigia palustis*	60 480 ± 4450	733 ± 364
*Lythrum salicaria*	78 100 ± 68 300	19 533 ± 6163
*Mikania scandens*	380 ± 197	9567 ± 1874
*Phragmites australis*	0	3500 ± 1077
*Pilea pumila*	2230 ± 1540	0
*Symphyotrichum puniceum*	2067 ± 980	317 ± 120
*Typha* spp.^a^	67 ± 49	650 ± 245
Total density	165 300 ± 74 850	34 933 ± 4823
Species richness	13.8 ± 0.5	7.8 ± 0.8
Total species	27	15
EM		
*Juncus effusus*	13 630 ± 6510	1927 ± 641
*Lindernia dubia*	950 ± 360	33 ± 17
*Ludwigia palustris*	8630 ± 6700	650 ± 255
*Lythrum salicaria*	134 800 ± 49 700	8150 ± 2564
*Mikania scandens*	1300 ± 620	4683 ± 1891
*Phragmites australis*	17 ± 17	3683 ± 1096
*Pilea pumila*	3770 ± 2300	33 ± 17
*Typha* spp.^a^	267 ± 267	683 ± 174
Total density	176 480 ± 47 490	20 317 ± 4640
Species richness	15.5 ± 2	8.8 ± 0.3
Total species	35	21
SM		
*Juncus acuminatus*	16 180 ± 10 800	17 ± 14
*Lindernia dubia*	13 280 ± 4560	183 ± 90
*Ludwigia palustris*	19 300 ± 10 100	950 ± 450
*Lythrum salicaria*	110 000 ± 34 470	2783 ± 564
*Mikania scandens*	180 ± 180	2267 ± 1074
*Mimulus* spp.^a^	2990 ± 1240	50 ± 28
*Phragmites australis*	0	1583 ± 740
*Symphyotrichum puniceum*	9970 ± 5190	100 ± 42
Total density	147 400 ± 61 600	8233 ± 2570
Species richness	17.2 ± 0.9	6.3 ± 0.9
Total species	34	12

^a^*Mimulus* species—*M. alatus, M. ringens. Typha* species—*T. angustifolia, T. latifolia.*

The 3–6 cm depth samples collected in 1998 showed substantial declines compared with surface 0–3 cm samples (Table 5). In 2011, however, values for 3–6 cm samples exceeded 0–3 cm ones, and total species was nearly double.

**Table 5:** **Comparisons of seed banks in 0–3 and 3–6 cm depth samples for March 1999 and 2011 at NM, EM and SM midpoint locations.** Data are density m^−2^ (*x* ± SE), species richness (*x* ± SE/sample) and total species. *n* = 3 for 1999 and *n* = 6 for 2011.

	Density	Species richness	Total species
1999			
NM 0–3 cm	382 080 ± 38 649	28.7 ± 3.0	58
NM 3–6 cm	14 100 ± 1369	7.0 ± 6.0	18
EM 0–3 cm	161 560 ± 32 970	29.1 ± 1.2	57
EM 3–6 cm	11 733 ± 1543	12.3 ± 1.2	24
SM 0–3 cm	305 460 ± 49 903	28.7 ± 1.5	56
SM 3–6 cm	14 433 ± 13 805	4.7 ± 2.6	10
2011			
SM 0–3 cm	29 467 ± 4880	8.0 ± 0.4	14
SM 3–6 cm	40 600 ± 9041	10.0 ± 1.0	23

#### Invasive species

The three important invasive species were *Lythrum*, *Phalaris arundinacea* and *Phragmites* (Table 6). Both *Lythrum* and *Phalaris* were more important in early years. In 2011, *Lythrum* occurred in the vegetation only in NM locations. *Phragmites*, in contrast, increased in importance in both the seed bank and vegetation at all sites.

**Table 6:** **Maximum seed bank densities and vegetation frequencies (FQ %) for invasive species from 1995–99 and 2011.** Data are density m^−2^ (*x* ± SE) and vegetation frequencies are % (based on all sites and locations) for 1995–98 and for 2011. For 1995–98, seed bank *n* = 216, vegetation *n* = 324; and for 2011, seed bank *n* = 54, vegetation *n* = 81.

	1995–99		2011	
Species	Seed bank density	Vegetation (FQ %)	Seed bank density	Vegetation (FQ %)
*Lythrum salicaria*	207 900 ± 30 760	89	29 280 ± 19 300	14^a^
*Phalaris arundinacea*	567 ± 454	47	17 ± 14	3^a^
*Phragmites australis*	133 ± 133	28	10 380 ± 2974	98

^a^Present only in NM plots.

Colonization by invasive species varied temporally and spatially. At the SM site, *Lythrum* was already present at all locations by August 1995 (Fig. 3C) [see Additional Information]. It quickly achieved high cover in the upland edge plots, but then was eliminated due to shading by woody canopy species. It persisted in midpoint plots over the entire sampling period, but at low values. During the 17-year sampling period, *Phragmites* did not appear in SM channel edge plots, and took several years to colonize other locations (Fig. 3D). It reached maximum cover in midpoint plots, and persisted at levels between 20 and 40 % in upland edge plots.

The 1999 clipping experiment found no significant effect of *Lythrum* cover on the total number of species (*P* = 0.42) or of cover species (*P* = 0.68) regardless of treatment (Licsko 2000).

## Discussion

This follow-up of the 1995–99 study of a created tidal freshwater wetland (Leck 2003; Leck and Leck 2005) provides several important insights. These relate to the continuing dispersal potential of the adjacent tidal Delaware River and to diversity, vegetation development and relationship to the seed bank, species persistence in the seed bank and the vegetation, and impact of invasive species.

### Dispersal and diversity

The dispersal of plant propagules into the created wetland area where no remnant wetland seed bank existed was immediate, creating diverse communities. These differed among sites, related in part to construction history, and with location, attributed to inundation regimes and seed deposition patterns (Leck 2003; M. A. Leck, pers. observ.). The numbers of species and the families they represented were high (Table 1), and of the most diverse families, the number of Cyperaceae species (33) in seed bank samples was greater than those recorded in 103 other studies reviewed by Leck and Schütz (2005). The initial planted macrophytes (12) contributed very little to overall diversity (194 species) (Leck and Leck 2005).

Propagules were of regional and local origins mostly carried by the Delaware River which extends 322 km north of the created wetland and Crosswicks Creek which flows from the southeast ∼37 km through tidal marsh and swampland (Leck 2003). Although Crosswicks Creek meets the Delaware ∼2 km downstream of the created wetland, lateral tidal movement in the Delaware River estuary is as much as 16.7 km (Sharp 1988).

Evidence for continuing dispersal was the eight new species added to the seed bank inventory and five to the vegetation in 2011, and the latter were all woody species (Appendix 1). Also, observations elsewhere in the created wetland revealed other colonizing species including, e.g., *Cardamine impatiens*, *Polygonum perfoliatum*, *Sagittaria subulata*, *Samolus floribundus* and *Strophostyles helvola*. The first two are incipient invasives in upland areas.

Although not studied for most species, estimates of dispersal modes can be determined from species seed characteristics, including size and appendages. Thus, ∼20 vs. 34 % of species (based on Leck 2003 vs. Appendix 1) have hairs or wings suitable for wind dispersal; 10 vs. 22 % are eaten or cached by animals; and 1 vs. 1.5 % have explosive fruits. The remaining 70 vs. 44 % are likely to be primarily dispersed by water. Hydrochory, as a primary dispersal mode, was noted by Neff and Baldwin (2005) for an Anacostia River tidal freshwater marsh, and it was also important in other wetlands (e.g. Middleton 2000; Vogt *et al*. 2007; Gurnell *et al.* 2008). Propagules, especially during late autumn and early winter 1995–97, were distributed in seed-containing wrack that was deposited away from the channel edges, but annual floods were also a potential source. Once vegetation developed, wrack appeared less (M. A. Leck, pers. observ.). No new species appeared in midpoint locations in 2011, suggesting that established vegetation limited dispersal.

Many species, however, have more than one mode of dispersal. Even those with appendages for aerial dispersal, e.g. *Acer* spp. and *Salix* spp., can be secondarily dispersed by water (M. A. Leck, pers. observ.) and others, such as *Juncus* and *Mimulus* spp., have small dust-like seeds that can float and be carried by wind. *Impatiens* has explosive fruits, and seeds that are cached by small rodents and that can float for several months (Parker and Leck 1985; M. A. Leck, pers. observ.), all effective modes of dispersal. For some, strategies may include release over a long period of time, including during winter, e.g. *Lythrum* (Klips and Peñalosa 2003). Furthermore, even species primarily dispersed by animals can be dispersed by water if fruit and/or seed structures provide buoyancy.

The increased animal dispersal in 2011 is probably related to the occurrence of avian-dispersed, woody species, such as *Morus* and *Vitis* spp., along the upland edge. Although actual evidence for animal dispersal is limited, the presence of birds and turtles has been well documented, beginning in 1994 (C. F. Leck, pers. comm.), and both may transport viable seeds (e.g. Braun and Brooks 1987; Mueller and van der Valk 2002; Soons *et al*. 2008; Wongsriphuek *et al*. 2008; Brochet *et al*. 2010; Kimmons and Moll 2010).

In addition to seeds, seedlings, such as those of *Lythrum* and *Pontederia cordata*, may float (e.g. Leck and Outred 2008). Diverse seedlings may be carried on wrack and other floating debris, guided by wind, tides and/or water currents (M. A. Leck, pers. observ.). Water-dispersed plants and plant fragments, e.g. *H. multiflora* and *Ludwigia peploides*, have also been observed, especially after summer or autumn floods.

Multiple factors contributed to diversity and they included, in addition to the effective dispersal of seeds, seedlings and plant fragments, spatially and temporally diverse habitats. Habitats varied, e.g., in duration of inundation, winter scour pattern, initial vegetation development related to construction schedule, sedimentation, and woody cover at upland edge locations. Furthermore, species varied in tolerances to the changing features of the created wetland.

It can be assumed that diverse propagules, including rare species, will continue to be dispersed into the wetland. However, their future esblishment is not guaranteed as vegetation changes and sedimentation alter conditions.

### Seed bank and vegetation development

Beginning in 1994/1995, colonization was rapid (Leck 2003), creating communities that were more diverse, of different composition and with seed banks of much higher densities than a reference marsh ∼2 km distant that had been studied for 15 years (Leck and Leck 2005). The higher diversity compared with reference sites, similar to Baldwin (2004), can be explained by the proximity of the created wetland to the Delaware River (Fig. 1). These high initial density and diversity values, however, were not sustained, and although still higher than most reference marsh sites (Leck and Leck 2005), 2011 values were lower than those from 1995–99 (Tables 1, 3 and 4). Lower densities could be related to changes, especially an increase in *Phragmites* cover, that eliminated species and/or reduced *in situ* seed production, especially by *Lindernia*, *Ludwigia* and *Lythrum* (Table 3; Fig. 3D; Leck 2003), and that appeared to lessen wrack deposition. Only *Mikania* and *Phragmites* seed bank densities increased (Appendix 2).

**Fig. 4:**
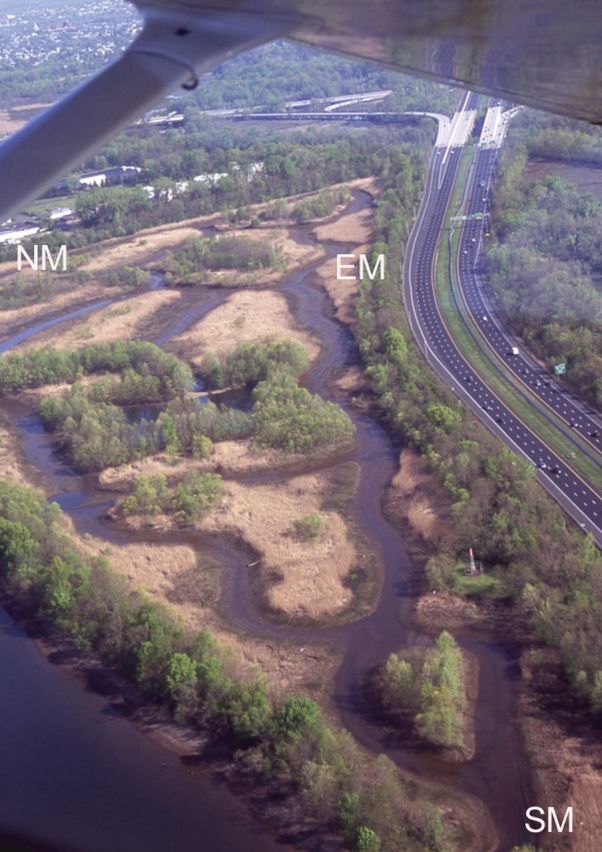
**Extent of *Phragmites* (light brown) during April 2010.** This aerial view of the created wetland was taken in early spring before herbaceous growth was visible; *Phragmites* thatch from 2009 growth is light brown. Also indicated is the east marsh (EM); only small portions of the south marsh (SM) and north marsh (NM) sites are visible.

With succession came changes in establishment characteristics. As the vegetation became denser, open habitats and small tide pool depressions were lost. The varied growth forms, an early feature related to the open nature of the wetland, ranged from creeping (*Ludwigia*), short upright (*Gratiola*, *Lindernia*), to tall (*J. effusus*, *Lythrum*), and, although continuing to be present in the seed bank, were generally lost from the vegetation except along channels and in places where disturbance created gaps. Herbivory was minimal and not noted in study plots; elsewhere grazing, primarily by Canada geese (*Branta canadensis*) on *Pontederia* and *Zizania*, was not observed after the first 2–3 years (C. F. Leck, pers. comm.). Ultimately, losses from the vegetation were associated with the increasing importance of *Phragmites*, especially away from channel edges. Establishment of woody species in tidal areas was limited. None was rooted in vegetation plots in 2011, but, although infrequent, woody plants (e.g. *Salix* spp.) were evident elsewhere (e.g. Fig. 4) [see Additional Information].

Sedimentation, although not measured directly, also appeared to alter establishment characteristics. Some sedimentation appeared to be related to erosion and slumping of channel edges, but external sources included both Crosswicks Creek and the Delaware River, which were sediment laden following heavy rains. Accumulation occurred in channels, which were colonized by *Nuphar* (M. A. Leck, pers. observ.), as well as in other areas. Other substrate features were also important. For example, at the SM site, individual *Lythrum* plants were frost-heaved during winter 2001 and died, and on one of the islands diversity in some monitoring spots was reduced to *Lythrum* with intervening spaces occupied almost exclusively by *Ludwigia* (e.g. in 1999, M. A. Leck, unpubl. data) [see Additional Information]. There, apparently, the packed gravelly substrate following construction and added sediment precluded the development of vegetation (Leck 2003; M. A. Leck, pers. observ.; Baldwin 2004).

The composition of both the seed bank and vegetation varied temporally. At 5 years, seed bank dominants that varied in relative importance with site were *J. effusus*, *Lindernia*, *Ludwigia* and *Lythrum*, and at 17 years they were *Lythrum*, *Mikania* and *Phragmites* (Table 3). The 17-year values reflect the continuing presence of *Lythrum* in the seed bank (see below) and vegetation, and contributions of *Phragmites* and *Mikania* to the seed bank as they became dominant in the vegetation. In the case of *J. effusus*, an EM dominant in 1995, its virtual loss from the vegetation by 1997 was rapid (Leck 2003), yet it was still important in the EM seed bank in 2011.

Dynamics varied with location (Fig. 3). At the time the study was begun in 1995, the channel edge at the SM site had no vegetation and was subsequently scoured clean of any wrack or dead plant remains during winter (M. A. Leck, pers. observ.); the decline in diversity in the midpoint plots was apparently due to the increasing importance of *Phragmites*; and the more gradual decline along the upland edge was probably related to both the presence of *Phragmites* and the shading canopy of woody species growing on the adjacent upland.

The behaviour of individual species was key to the vegetation changes observed, and distributions were related to innate species characteristics. The preference, for example, of *P. punctatum* for channel edge locations is related to its ability to tolerate inundation stresses, while *Impatiens* is better able to tolerate competition stress and shade at midpoint or upland edge locations (Fig. 3A and B; Parker and Leck 1985; Simpson *et al*. 1985). Neither species, however, appeared able to persist with the advance of *Nuphar* from the channel or *Phragmites* in midpoint or upland edge locations. Other species similarly waxed and waned.

### Persistence

Persistence in the vegetation, which varied among species, is easily demonstrated by observing changes in cover over time with site and location (e.g. Table 3; Fig. 3). For seed banks, persistence, which also showed considerable variability among species, was evaluated from species still present in June samples, and by comparing those in 3–6 cm samples with surface 1–3 cm samples (Leck 2003). June samples contained substantial numbers of seeds of *Lindernia*, *Ludwigia* and *Lythrum*, and of *Mikania* and *Phragmites* in 2011, indicating ability to persist at least for the short term (Table 4; Leck 2003). In 1999, seed bank dominants, besides *Lythrum*, included *Ludwigia* and, depending on site, *Lindernia*, *J. effusus* or *J. acuminatus*. Species germinating from 3–6 cm samples (Table 5; e.g. *Lindernia*, *Ludwigia*, *Lythrum*; Leck 2003) had been buried for some undetermined length of time. [Unfortunately, the 2011 depth samples, because of unexpected (and unexplained) high values (Table 5), could not be used to substantiate longevity.] However, all of these species are small seeded, a characteristic of species with a persistent seed bank strategy (Leck and Brock 2000; Fenner and Thompson 2005).

Despite difficulty in assigning longevity seed type (see Fenner and Thompson 2005), persistent seeds in soil, including species not found in the vegetation (Appendix 2), may contribute to future vegetation if suitable establishment conditions occur. The future presence of *Impatiens*, a transient seed bank species with seeds persisting for <1 year (Leck and Simpson 1987), will require areas not dominated by *Phragmites* where *Impatiens* can produce seeds each year, as found in the NM in 2011, and/or external sources of seeds coupled with seed dispersal.

### Invasive species

By 2011, vegetation changes, except along channel edges and limited parts of the NM site (also in limited areas of some islands), were related to the dominance of *Phragmites. Lythrum* and *Phalaris* had little impact on long-term vegetation patterns, and although *Lythrum* had established in all sites by August 1995 (Leck and Leck 2005; M. A. Leck, pers. observ.), it was eventually replaced by *Phragmites. Lythrum*, unlike *Phragmites*, did not reduce species diversity (Licsko 2000). Where *Phragmites* was not present, diverse communities were often present (M. A. Leck, pers. observ.) [see Additional Information].

The abundance of *Phragmites* in the seed bank was higher than for Chesapeake Bay brackish wetlands (284–698 m^−2^) and other studies noted by Baldwin *et al*. (2010). Moreover, the high densities in both March and June samples indicate high viability and persistence, and suggest that viable seeds are produced annually, a feature associated with the invasive genotype (Kettenring and Whigham 2009; Baldwin *et al*. 2010; Kettenring *et al*. 2010; D. F. Whigham, pers. comm.). North marsh specimens, examined in 2012, were of the invasive genotype (M. A. Leck, pers. observ.; Swearingen and Saltonstall 2010).

As *Phragmites* continues to expand, small relict communities that are *Phragmites* free both within the created wetland and regionally will be important sources of propagules for sustaining species (e.g. van der Valk *et al*. 2009).

Although *Phragmites* was slow to become an important part of the created wetland vegetation (e.g. Fig. 3D; Leck 2003), its presence was noted as early as 1994 in various areas with colonization occurring intermittently over the entire wetland in subsequent years (M. A. Leck, pers. observ.), suggestive of high genetic diversity (Kettenring *et al.* 2010). Now, it is estimated to cover ∼80 % of the created wetland (Fig. 4) [see Additional Information]. Wetland hydrologic design, which provided conditions favourable for the establishment of many species while gaps and low-nutrient sediment (sand and gravel) were present, was, nonetheless, a wetland disturbance, available for establishment by *Phragmites*. *Phragmites* appears to be a keystone species in this wetland, and even persistent seed bank species and newly dispersed propagules have little chance of contributing to vegetation development unless other disturbances create gaps in the dense *Phragmites* patches.

One of the design objectives for this wetland was creation of wildlife habitat (M. Kaminsky, pers. comm.), and in the first year >100 bird species and other animals, including box turtles (*Terrapene carolina*) and muskrats (*Ondatra zibethicus*), were observed there. However, as with vegetation, animal diversity has declined, probably associated with the increase in *Phragmites* and decrease in forage species (C. F. Leck, pers. comm.). Any attempts now to reduce *Phragmites* would be very costly (B. Hawkinson and N. J. Dott, pers. comm.). Unfortunately, long-term monitoring, a highly desirable feature for restoration sites, was not a part of the mitigation plan, and an impact statement prepared during 1995 only documented initial vegetation coverage, and actually indicated that neither *Lythrum* nor *Phragmites* would become problems (Marble and Company 1998). Certainly, the success of wetland creation cannot be assessed in 3–5 years (see Kellogg and Bridgham 2002; Baldwin 2004) and, then, neither can success be equated with composition similar to that of natural wetlands (Ehrenfeld 2000; Baldwin 2004). It is apparent that restoration plans should include initial and future goals, as well as realistic criteria for evaluation (see e.g. Simenstad *et al*. 2006; Baldwin *et al*. 2009).

## Conclusions and forward look

Colonization, driven by the availability of propagules transported by the nearby Delaware River, was immediate, within 1 year resulting in a highly diverse seed bank and vegetation and very high seed bank densities. Input of seeds continued over the 17 years of the study, as new species were observed in 2011. With time, however, densities and species diversity declined as establishment characteristics changed, related to sedimentation and vegetation development in areas that were occupied by *Phragmites*. Vegetation development reduced seed production of many early colonizing, low-growing species, resulting in lower total seed bank densities at all the locations at all sites in 2011. As the vegetation changed, species differed in persistence but were often present in the seed bank for longer than in the vegetation. In some cases, initial site colonization differences were still apparent in 2011 seed bank samples. At the end of the 17-year study period, species totals and richness were less, and most species had lower seed bank densities and cover values. The only exceptions were *Mikania* and *Phragmites*, which were well represented in 2011 in both the seed bank and vegetation. Invasive species were also dynamic temporarily; *Lythrum*, important between 1995 and 2002, did not continue as a dominant, and *Phragmites* became the key species especially in midpoint and upland edge locations.

Although it appears that vegetation progress (succession) in this created tidal freshwater wetland is now determined by *Phragmites*, future monitoring will permit a better understanding of the continued expansion of this species into small areas that are still *Phragmites* free. The high seed bank density and persistence of *Phragmites*, compared with published studies, suggests that studies exploring germination viability and longevity might provide insights into its invasiveness. It is important that future studies explore how restoration failed to maintain the high initial species diversity or develop the characteristic vegetation of nearby reference marsh sites. Insights from this restoration should be applied to future ones. Future seed bank studies may also determine the longevity of seeds, suggestive of their importance in restoration here and elsewhere.

## Additional information

The following additional information is available in the online version of the article –

File 1: Addendum with photographs of the created wetland sites from 1994 to 2011.

## Sources of funding

A grant from the New Jersey Water Resources Research Institute supported Karen Licsko's study of the impact of *Lythrum salicaria* on biodiversity. All other support was provided by Rider University.

## Conflicts of interest statement

None declared.

## Supplementary Material

Additional Information

## Appendix

**Appendix 1:** **Species (families) emerging from soil samples and/or present in vegetation plots in 2011.** Species seed bank values for March samples are maximum densities (*x* ± SE); site and location for maxima are indicated. Location frequencies (FQ) are based on *n* = 18 (ch, mi) or *n* = 16 (up) for seed bank data and *n* = 27 for vegetation plots. Sites are NM, EM and SM (see Fig. 1), and locations are channel edge (ch), midpoint (mi) and upland edge (up). Species not recorded in 1995–99 (Leck 2003) are noted with an asterisk. A ‘w’ indicates woody species, including vines. Nomenclature follows USDA (2012).

Species	Seed bank					Vegetation		
	Maximum	Site location	FQ ch	FQ mi	FQ up	FQ ch	FQ mi	FQ up
*Acer negundo* (Aceraceae) w								7.4
*Acer rubrum** (Aceraceae) w								3.7
*Acer saccharinum* (Aceraceae) w								22.2
*Agrostis hyemalis** (Poaceae)	17 ± 14	E ch	5.6					
*Ailanthus altissima* (Simaroubaceae) w	25 ± 16	S up			6.3			
*Alnus* sp. (Betulaceae) w								11.1
*Amaranthus cannabinus* (Amaranthaceae)	117 ± 68	E ch	27.8	5.6		42.3		
*Ambrosia artemisiifolia* (Asteraceae)	33 ± 17	S ch	11.1					
*Amorpha fruticosa* (Fabaceae) w	17 ± 14	E ch	5.6					33.3
*Artemisia annua** (Asteraceae)	17 ± 14	N ch	5.6					
*Artemisia vulgaris* (Asteraceae)	17 ± 14	N ch	5.6					
*Betula nigra* (Betulaceae) w								3.7
*Bidens laevis* (Asteraceae)	17 ± 14	E mi		5.6				
*Boehmeria cylindrica* (Urticaceae)	67 ± 27	N ch	22.2					
*Carex scoparia* (Cyperaceae)	17 ± 14	N up			6.3			
*Carex* sp. 1 (Cyperaceae)	33 ± 27	N up			12.5			
*Carex* sp. 2 (Cyperaceae)	67 ± 40	E mi		11.1	12.5			
*Chenopodium ambrosioides* (Chenopodiaceae)	17 ± 14	S ch	5.6					
*Clematis terniflora* (Ranunculaceae) w	17 ± 14	N mi			6.3			44.4
*Conyza canadensis* (Asteraceae)	17 ± 14	N mi		5.6				
*Cornus amomum** (Cornaceae) w								18.5
*Cuscuta gronovii* (Cuscutaceae)	117 ± 80	N up		5.6	12.5			
*Cyperus bipartitus* (Cyperaceae)	17 ± 14	N mi		5.6				
*Cyperus odoratus* (Cyperaceae)	33 ± 17	E mi	5.6	16.7				
*Cyperus* sp. 1 (Cyperaceae)	25 ± 17	S up		11.1	6.3			
*Cyperus* sp. 2 (Cyperaceae)						7.7		
*Cyperus strigosus* (Cyperaceae)	75 ± 17	S up		22.2	31.3			
*Dichanthelium clandestinum* (Poaceae)	17 ± 14	E ch	5.6					
*Echinochloa crus-galli* (Poaceae)	50 ± 28	S ch	27.8			3.8		
*Echinochloa muricata** (Poaceae)	17 ± 14	E ch	5.6					
*Eclipta prostrata* (Asteraceae)	17 ± 14	E mi		5.6				
*Eleocharis engelmannii* (Cyperaceae)	267 ± 141	E ch	22.2					
*Elodea* sp*.** (Hydrocharitaceae)						11.1		
*Erigeron* sp. (Asteraceae)	17 ± 14	E ch	5.6					
*Eupatorium serotinum* (Asteraceae)	50 ± 41	E ch	5.6	5.6	12.5	3.8		
*Euthamia graminifolia* (Asteraceae)	17 ± 14	S ch	5.6					
*Fraxinus* sp. (Oleaceae) w	17 ± 14	N ch	5.6					7.4
*Gratiola neglecta* (Scrophulariaceae)	267 ± 98	E ch	27.8	27.8	12.5			
*Helenium autumnale* (Asteraceae)	17 ± 14	S ch	5.6			3.8		
*Heteranthera multiflora* (Pontederiaceae)						7.7		
*Heteranthera* sp. *(multiflora?)*(Pontederiaceae)	50 ± 41	S ch	5.6	11.1				
*Hypericum mutilum* (Clusiaceae)	50 ± 18	N ch, E mi	22.3	16.7				
*Ilex verticillata** Aquifoliaceae) w								3.7
*Impatiens capensis* (Balsaminaceae)	200 ± 89	N up		11.1	31.3	3.8	4.2	14.8
*Juncus acuminatus* (Juncaceae)	600 ± 442	N up		44.4	50			
*Juncus effusus* (Juncaceae)	15 400 ± 3078	E mi	11.1	38.9	62.5			
*Junus secundus/tenuis)* (Juncaceae)	33 ± 27	E ch	11.1	5.6	6.3			
*Leersia oryzoides* (Poaceae)	17 ± 14	E ch, E mi	5.6	5.6		7.7	4.2	3.7
*Lindernia dubia* (Scrophulariaceae)	525 ± 236	S up	38.9	50	62.5	2.7		
*Lonicera japonica** (Caprifoliaceae) w								3.7
*Ludwigia palustris* (Onagraceae)	14 567 ± 3166	E up	61.1	100	87.5	3.8		
*Lycopus americanus* (Lamiaceae)	17 ± 14	E mi	5.6	5.6				
*Lycopus europaeus* (Lamiaceae)	17 ± 14	S ch	11.1					
*Lythrum salicaria* (Lythraceae)	29 283 ± 19 300	N up	77.8	18	93.8	3.8	25	14.8
*Microstegium vimineum* (Poaceae)	17 ± 14	E ch	5.6					
*Mikania scandens* (Asteraceae)	14 600 ± 3299	S mi	55.6	100	100	2.7	58.3	14.8
*Mimulus alatus* (Scrophulariaceae)						3.8		
*Mimulus ringens* (fl) (Scrophulariaceae)	33 ± 27	S mi		5.6				
*Mimulus* sp*.* (Scrophulariaceae)	400 ± 246	S up		11.1	25			
*Morus* sp.* (Moraceae) w	100 ± 27	S up			31.3			
*Myosotis* sp. (Boraginaceae)						2.7		
*Nuphar lutea* (Nymphaeaceae)						30.8	4.2	
*Oenothera biennis* (Onagraceae)	17 ± 14	S ch	5.6					
*Peltandra virginica* (Araceae)						3.8	16.7	3.7
*Penthorum sedoides* (Crassulaceae)	33 ± 27	E up			18.8			
*Phalaris arundinacea* (Poaceae)	17 ± 14	S ch	5.6					7.4
*Phragmites australis* (Poaceae)	10 383 ± 2974	E mi	72.2		3.8	19.2	87.5	85.2
*Phytolacca americana* (Phytolaccaceae)	175 ± 74	S up		5.6	25			
*Pilea pumila* (Urticaceae)	1800 ± 661	N up	33.3		31.3	15.4	4.2	18.5
*Plantago rugelii* (*major*) (Plantaginaceae)	17 ± 14	S ch	5.6					
*Platanus occidentalis* (Platanaceae) w	33 ± 27	E up		11.1	6.3			14.8
*Polygonum cespitosum* (Polygonaceae)	17 ± 14	N,E up			12.5			
*Polygonum hydropiper* (Polygonaceae)	66 ± 40	N mi	5.6					
*Polygonum hydropiperoides* (Polygonaceae)	117 ± 80	S ch	27.8					
*Polygonum punctatum* (Polygonaceae)	350 ± 119	S ch	55.6			84.6	4.2	
*Polygonum sagittatum* (Polygonaceae)								3.7
*Pontederia cordata* (Pontederiaceae)						19.2		
*Populus* sp. (Salicaceae) w								3.7
*Potentilla* sp.* (Rosaceae)	17 ± 14	S up			6.3			
*Rhus typhina** (Anacardiaceae) w	33 ± 27	N up			18.8			3.7
*Robinia pseudoacacia* (Fabaceae) w	33 ± 27	N up			6.3			59.3
*Rubus* sp.* (Rosaceae) w	17 ± 14	E up			6.3			
*Salix nigra* (Salicaceae) w								29.6
*Sium suave* (Apiaceae)						2.7	8.3	7.4
*Symphyotrichum puniceum* (Asteraceae)	433 ± 98	N mi	33.3	61.1	31.3	19.2	4.2	7.4
*Typha angustifolia* (Typhaceae)						2.7	12.5	14.8
*Typha latifolia* (Typhaceae)						30.8	12.5	
*Typha* spp. (Typhaceae)	2050 ± 854	N mi	11.1		62.5			
*Ulmus* sp.* Ulmaceae) w								3.7
umbel (Apiaceae)	17 ± 14	N mi		5.6				
*Vernonia noveboracensis* (Asteraceae)						8.3		3.7
*Veronica peregrina* (Scropulariaceae)	50 ± 41	N ch	5.6					
*Vitis* sp.* (Vitaceae) w	50 ± 19	S up			12.5			18.5
*Zizania aquatica* (Poaceae)						2.7		

## Appendix

**Appendix 2:** **Comparisons of sites and locations for seed bank and vegetation in 2011.** For sites see Fig. 1. Data are seed bank density m^−2^ (*x* ± SE), % cover (*x* ± SE), species richness (*x* ± SE) and total species. Shown are seed bank species ≥100 m^−2^, and vegetation species ≥10 % cover as well as *N. lutea*. Upland edge species are only those rooted in the plots; woody overhanging species are not included. Locations: ch, channel edge; mi, midpoint; up, upland edge.

NM	Seed bank	Vegetation	EM	Seed bank	Vegetation	SM	Seed bank	Vegetation
ch			ch			ch		
*Amaranthus cannabinus*	33 ± 27	8.9 ± 3.1	*Amaranthus cannabinus*	117 ± 68	6.7 ± 2.6	*Elodea* sp.	0	25.6 ± 12.1
*Gratiola neglecta*	200 ± 105	0	*Eleocharis engelmannii*	267 ± 141	0	*Ludwigia palustris*	100 ± 52	0
*Lindernia dubia*	483 ± 145	1.8 ± 1.7	*Gratiola neglecta*	267 ± 98	0	*Lythrum salicaria*	183 ± 93	0
*Ludwigia palustris*	1517 ± 567	1.7 ± 1.7	*Lindernia dubia*	183 ± 85	0	*Nuphar lutea*	0	51.9 ± 11.5
*Lythrum salicaria*	2800 ± 1279	2.2 ± 2.2	*Ludwigia palustris*	417 ± 131	0	*Peltandra virginica*	0	9.4 ± 8.8
*Mikania scandens*	883 ± 280	6.1 ± 4.5	*Lythrum salicaria*	1050 ± 411	0	*Phragmites australis*	283 ± 102	0
*Nuphar lutea*	0	7.7 ± 5.7	*Nuphar lutea*	0	0	*Polygonum hydropiperoides*	117 ± 80	?
*Phragmites australis*	833 ± 264	16.1 ± 7.2	*Phragmites australis*	717 ± 281	0.6 ± 0 .6	*Polygonum punctatum*	350 ± 119	4.4 ± 2.4
*Polygonum punctatum*	117 ± 68	28.3 ± 8.5	*Polygonum hydropiperoides*	117 ± 80	?			
*Pontederia cordata*	0	7.2 ± 4.5	*Polygonum punctatum*	267 ± 103	62.2 ± 7.0			
*Symphyotrichium puniceum*	67 ± 45	14.4 ± 6.2						
*Typha* spp.	0	13.9 ± 4.5						
Total density	7650 ± 1823		Total density	4117 ± 1354		Total density	1633 ± 590	
Species/sample	8 ± 1.5	6.1 ± 0.7	Species/sample	9 ± 1.7	1.78 ± 0.3	Species/sample	6.2 ± 2.5	3.1 ± 0.5
Total species	22	21	Total species	25	5	Total species	18	11
**NM**	**Seed bank**	**Cover**	**EM**	**Seed bank**	**Cover**	**SM**	**Seed bank**	**Cover**
**mi**			**mi**			**mi**		
*Impatiens capensis*	100 ± 67	1.1 ± 1.1	*Juncus effusus*	15 400 ± 3078	0	*Lindernia dubia*	300 ± 123	0
*Juncus acuminatus*	450 ± 240	0	*Lindernia dubia*	117 ± 49	0	*Ludwigia palustris*	2517 ± 729	0
*Lindernia dubia*	450 ± 238	0	*Ludwigia palustris*	4117 ± 1235	0	*Lythrum salicaria*	5617 ± 1663	6.7 ± 5.4
*Ludwigia palustris*	1183 ± 391	0	*Lythrum salicaria*	21 417 ± 6317	0	*Mikania scandens*	14 600 ± 3299	25.8 ± 7.6
*Lythrum salicaria*	18 570 ± 5954	18.3 ± 6.4	*Mikania scandens*	9367 ± 1290	28.3 ± 12.7	*Phragmites australis*	5400 ± 1213	78.3 ± 5.2
*Mikania scandens*	13 533 ± 1089	41.1 ± 12.1	*Peltandra virginica*	0	12.2 ± 6.6	*Symphyotrichium puniceum*	200 ± 84	0
*Phragmites australis*	3683 ± 1043	53.9 ± 14.5	*Phragmites australis*	10 383 ± 2974	84.4 ± 2.3	*Typha* spp.	383 ± 124	0
*Polygonum hydropiperoides*	100 ± 67	?	*Pilea pumila*	300 ± 143	0			
*Symphyotrichium puniceum*	517 ± 142	1.7 ± 1.7	*Typha* spp*.*	1167 ± 314	4.4 ± 3.1			
*Typha* spp.	2050 ± 854	12.4 ± 7.2						
Total density	42 067 ± 7354		Total density	63 383 ± 4797		Total density	29 500 ± 4880	
Species/sample	11 ± 1.0	3.2 ± 0.7	Species/sample	11.8 ± 1.3	2 + 0.3	Species/sample	8 ± 0.4	2 ± 0.2
Total species	25	12	Total species	24	4	Total species	14	4
								
**NM**	**Seed bank**	**Cover**	**EM**	**Seed bank**	**Cover**	**SM**	**Seed bank**	**Cover**
**up**			**up**			**up**		
*Cuscuta gronoviii*	117 ± 80	0	*Juncus acuminatus*	100 ± 52	0	*Juncus acuminatus*	225 ± 129	0
*Impatiens capensis*	200 ± 89	13.9 + 6.8	*Juncus effusus*	14 233 ± 3669	0	*Juncus effusus*	300 ± 159	0
*Juncus acuminatus*	116 ± 53	0	*Lindernia dubia*	317 ± 112	0	*Lindernia dubia*	525 ± 236	0
*Juncus effusus*	2383 ± 1850	0	*Ludwigia palustris*	14 567 ± 3166	0	*Ludwigia palustris*	1680 ± 686	0
*Lindernia dubia*	333 ± 186	0	*Lythrum salicaria*	3233 ± 738	0	*Lythrum salicaria*	1680 ± 538	0
*Ludwigia palustris*	1450 ± 784	0	*Mikania scandens*	1117 ± 177	0	*Mikania scandens*	825 ± 145	0
*Lythrum salicaria*	29 283 ± 19 300	12.8 ± 6.1	*Phragmites australis*	7033 ± 1080	31.7 ± 7.3	*Mimulus* spp*.*	400 ± 245	0
*Mikania scandens*	5533 ± 1936	15 ± 8.2				*Morus* spp*.*	100 ± 27	0
*Phragmites australis*	683 ± 144	13.9 ± 4.9				*Phragmites australis*	2730 ± 698	42.2 ± 11.2
*Pilea pumila*	1800 ± 661	15 ± 6.6						
*Typha* spp.	617 ± 219	11.1 ± 5.1						
Total density	43 317 ± 19 254		Total density	41 450 ± 4041		Total density	9130 ± 2140	
Species/sample	11.2 ± 0.7	6.3 ± 0.8	Species/sample	8.8 ± 0.5	3.9 ± 0.4	Species/sample	12 ± 1.1	4.3 ± 0.4
Total species	22	20	Total species	20	14	Total species	20	12
Woody species	3	5	Woody species	2	13	Woody species	4	11

## References

[PLS050C1] Baldwin AH (2004). Restoring complex vegetation in urban settings: the case of tidal freshwater marshes. Urban Ecosystems.

[PLS050C2] Baldwin AH, Hammerschlag RS, Cahoon DR, Gerardo M, Perillo E, Wolanski E, Cahoon DR, Brinson MM (2009). Evaluation of restored tidal freshwater wetlands. Coastal wetlands: an integrated ecosystem approach.

[PLS050C3] Baldwin AH, Kettenring KM, Whigham DF (2010). Seed banks of *Phragmites australis*-dominated brackish wetlands: relationships to seed viability, inundation, and land cover. Aquatic Botany.

[PLS050C4] Braun J, Brooks GR (1987). Box turtles (*Terrapene carolina*) as potential agents for seed dispersal. American Midland Naturalist.

[PLS050C5] Brochet A-L, Guillemain M, Gauthier-Clerc M, Fritz H, Green AJ (2010). Endozoochory of Mediterranean aquatic plant seeds by teal after a period of desiccation: determinants of seed survival and influence of retention time on germinability and viability. Aquatic Botany.

[PLS050C6] Brower JE, Zar JH, von Ende CN (1998). Field and laboratory methods for general ecology.

[PLS050C7] Diggory ZE, Parker VT (2010). Seed supply and revegetation dynamics at restored tidal marshes, Napa River, California. Restoration Ecology.

[PLS050C8] Ehrenfeld JG (2000). Evaluating wetlands within an urban context. Ecological Engineering.

[PLS050C9] Fenner M, Thompson K (2005). The ecology of seeds.

[PLS050C10] Gurnell A, Thompson K, Goodson J, Moggridge H (2008). Propagule deposition along river margins: linking hydrology and ecology. Journal of Ecology.

[PLS050C11] Kellogg CH, Bridgham SD (2002). Colonization during early succession of restored freshwater marshes. Canadian Journal of Botany.

[PLS050C12] Kettenring KM, Whigham DF (2009). Seed viability and seed dormancy of non-native *Phragmites australis* in suburbanized and forested watersheds of the Chesapeake Bay, USA. Aquatic Botany.

[PLS050C13] Kettenring KM, McCormick MK, Baron HM, Whigham DF (2010). *Phragmites australis* (common reed) invasion in the Rhone River subestuary of the Chesapeake Bay: disentangling the effects of foliar nutrients, genetic diversity, patch size, and seed viability. Estuaries and Coasts.

[PLS050C14] Kimmons JB, Moll D (2010). Seed dispersal by Red-eared Sliders (*Trachemys scripta elegans*) and Common Snapping Turtles (*Chelydra serpentina*). Chelonian Conservation and Biology.

[PLS050C15] Klips RA, Peñalosa J (2003). The timing of seed fall, innate dormancy, and ambient temperature in *Lythrum salicaria*. Aquatic Botany.

[PLS050C16] Leck MA (2003). Seed-bank and vegetation development in a created tidal freshwater wetland on the Delaware River, Trenton, New Jersey, USA. Wetlands.

[PLS050C17] Leck MA, Brock MA (2000). Ecological and evolutionary trends in wetlands: evidence from seeds and seed banks in New South Wales, Australia and New Jersey, USA. Plant Species Biology.

[PLS050C18] Leck MA, Crain CM, Barendregt A, Whigham DF, Baldwin AH (2009). Northeastern North America case studies—New Jersey and New England. Tidal freshwater wetlands.

[PLS050C19] Leck MA, Leck CF (2005). Vascular plants of a Delaware River tidal freshwater wetland and adjacent terrestrial areas: seed bank and vegetation comparisons of reference and constructed marshes and annotated species list. Journal of the Torrey Botanical Society.

[PLS050C20] Leck MA, Outred HA, Leck MA, Parker VT, Simpson RL (2008). Seedling natural history. Seedling ecology and evolution.

[PLS050C21] Leck MA, Schütz W (2005). Regeneration of Cyperaceae, with particular reference to seed ecology and seed banks. Perspectives in Plant Ecology, Evolution and Systematics.

[PLS050C22] Leck MA, Simpson RL (1987). The seed bank of a freshwater tidal wetland: turnover and relationship to vegetation change. American Journal of Botany.

[PLS050C23] Leck MA, Baldwin AH, Parker VT, Schile L, Whigham DF, Barendregt A, Whigham DF, Baldwin AH (2009). Plant communities of tidal freshwater wetlands of the continental USA and southeastern Canada. Tidal freshwater wetlands.

[PLS050C24] Licsko K (2000). *Removal of the invasive exotic*, *Lythrum salicaria (purple loosestrife): impact on plant diversity in the constructed wetland at the Hamilton/Trenton tidal freshwater marsh*.

[PLS050C25] **Marble and Company** (1998). *Post-construction wetland monitoring report*, *Trenton Complex mitigation site*, *Mercer County*, *New Jersey*.

[PLS050C26] Middleton B (2000). Hydrochory, seed banks, and regeneration dynamics along the landscape boundaries of a forested wetland. Plant Ecology.

[PLS050C27] Mueller MH, van der Valk AG (2002). The potential role of ducks in wetland seed dispersal. Wetlands.

[PLS050C28] Neff KP, Baldwin AH (2005). Seed dispersal into wetlands: techniques and results from a restored tidal freshwater marsh. Wetlands.

[PLS050C29] Parker VT, Leck MA (1985). Relationships of seed banks to plant distribution patterns in a freshwater tidal wetland. American Journal of Botany.

[PLS050C30] Rhoads AF, Klein WM (1993). The vascular plants of Pennsylvania: annotated checklist and atlas.

[PLS050C31] Sharp JH, Bryant TL, Penock JR (1988). Dynamics. The Delaware estuary: rediscovering a forgotten resource.

[PLS050C32] Simenstad C, Reed D, Ford M (2006). When is restoration not? Incorporating landscape-scale processes to restore self-sustaining ecosystems in coastal wetland restoration. Ecological Engineering.

[PLS050C33] Simpson RL, Leck MA, Parker VT (1985). The comparative ecology of *Impatiens capensis* Meerb. (Balsaminaceae) in central New Jersey. Bulletin of the Torrey Botanical Club.

[PLS050C34] Snyder DB (2010). Special plants of New Jersey.

[PLS050C35] Soons MB, van der Vlugt C, van Lith B, Hell GW, Klaassen M (2008). Small seed size increases the potential for dispersal of wetland plants by ducks. Journal of Ecology.

[PLS050C36] Swearingen J, Saltonstall K (2010). http://www.nps.gov/plants/alien/pubs/index.ht.

[PLS050C37] **USDA NRCS (United States Department of Agriculture, Natural Resources Conservation Service)** (2012). http://plants.usda.gov.

[PLS050C38] van der Valk AG, Toth LA, Gibney EB, Mason DH, Wetzel PR (2009). Potential propagule sources for reestablishing vegetation on the floodplain of the Kissimmee River, Florida, USA. Wetlands.

[PLS050C39] Vogt K, Rasran L, Jensen K (2007). Seed deposition in drift lines: opportunity or hazard for species establishment?. Aquatic Botany.

[PLS050C40] Whigham DF, Barendregt A, Whigham DF, Baldwin AH (2009). Primary production in tidal freshwater wetlands. Tidal freshwater wetlands.

[PLS050C41] Whigham DF, Simpson RL (1975). Ecological studies of the Hamilton Marshes, progress report for the period June 1974–January 1975.

[PLS050C42] Wongsriphuek C, Dugger BD, Bartuszevige AM (2008). Dispersal of wetland plant seeds by mallards: influence of gut passage on recovery, retention, and germination. Wetlands.

