# Supplementary material for: Dispersal potential of a tidal river and colonization of a created tidal freshwater marsh
Source: AoB Plants. 2012 Dec 21;5:pls050. doi: 10.1093/aobpla/pls050 (PMC4104633; doi:10.1093/aobpla/pls050)
Supplement: Additional Information [file supp_5_pls050_index.html]

Additional Information 

# Dispersal potential of a tidal river and colonization of a created tidal freshwater marsh

## Additional Information

**Files in this Data Supplement:**

- Additional Information - pdf file
