# Supplementary material for: Dispersal potential of a tidal river and colonization of a created tidal freshwater marsh
Source: AoB Plants. 2012 Dec 21;5:pls050. doi: 10.1093/aobpla/pls050 (PMC4104633; doi:10.1093/aobpla/pls050)
Supplement: Additional Information [file supp_pls050_pls050supp.pdf]

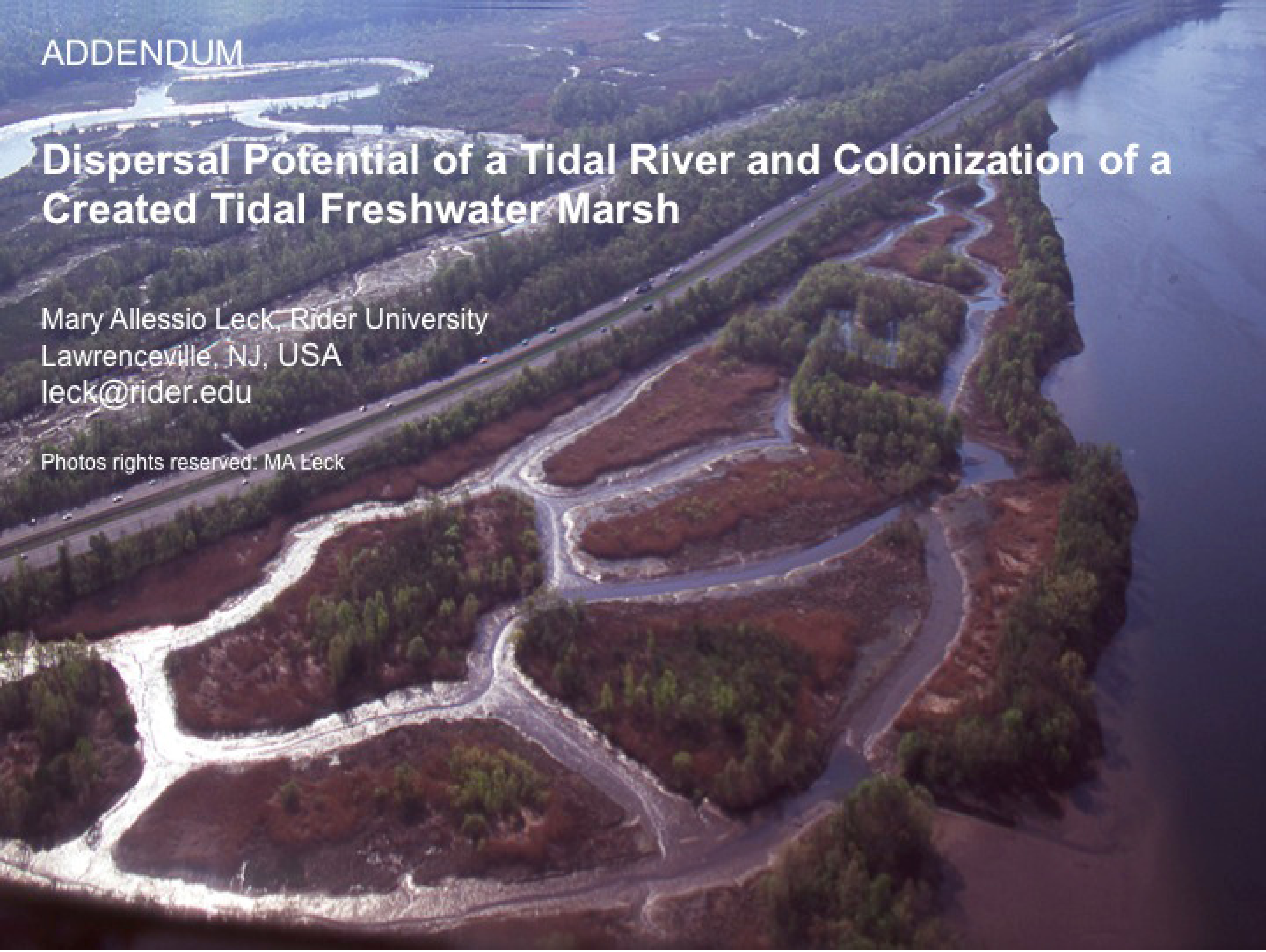
ADDENDUM
Dispersal Potential of a Tidal River and Colonization of a Created Tidal Freshwater Marsh
Mary Allessio Leck, Rider University
Lawrenceville, NJ, USA
leck@rider.edu
Photos rights reserved: MA Leck

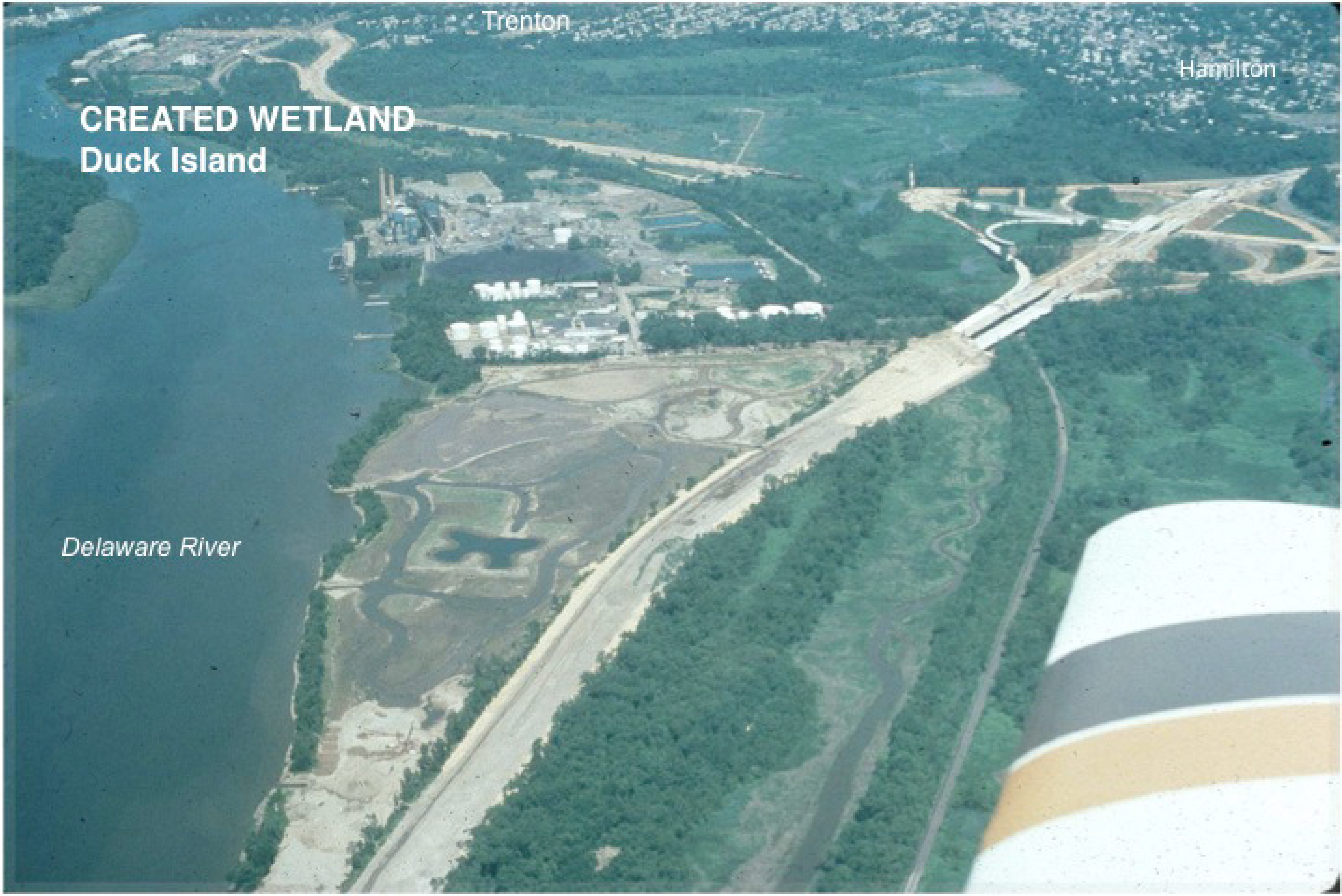

April 1994 –courtesy Joseph Schmeltz

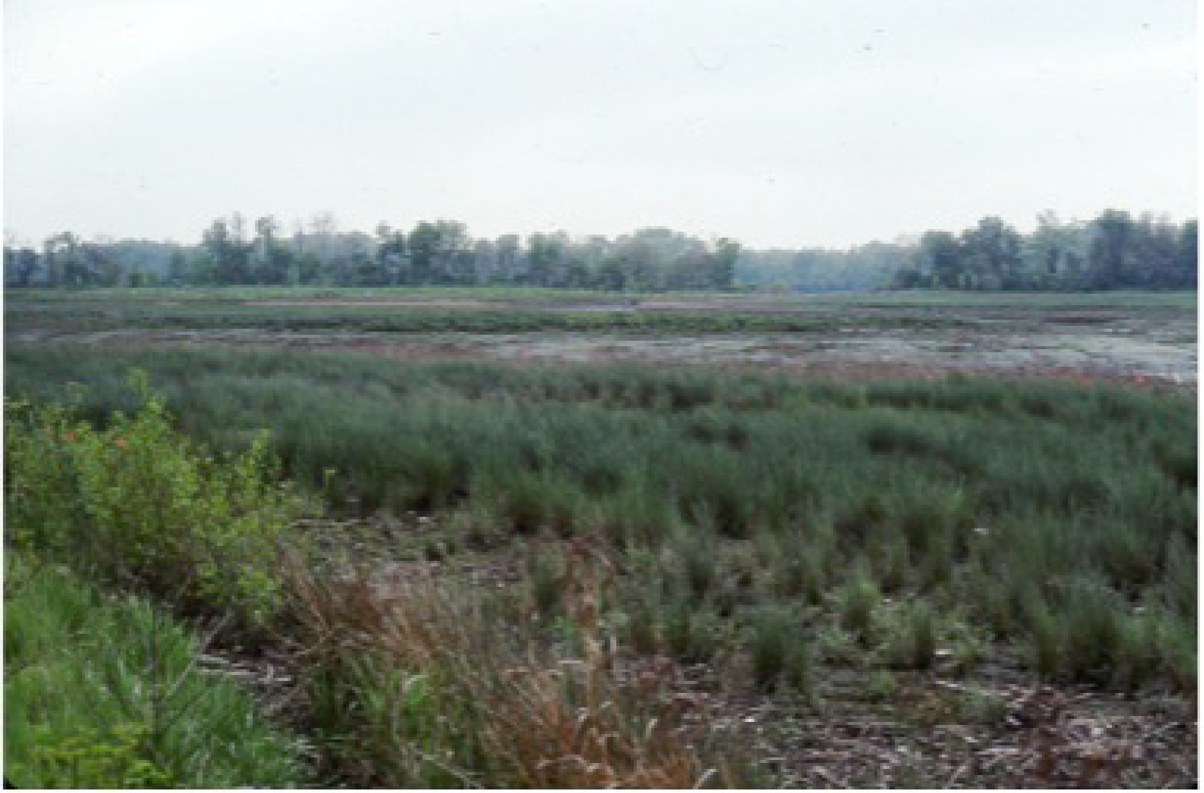

6 May '95
Juncus effusus

East Marsh

15 Aug '95
Lythrum salicaria
(flowering)

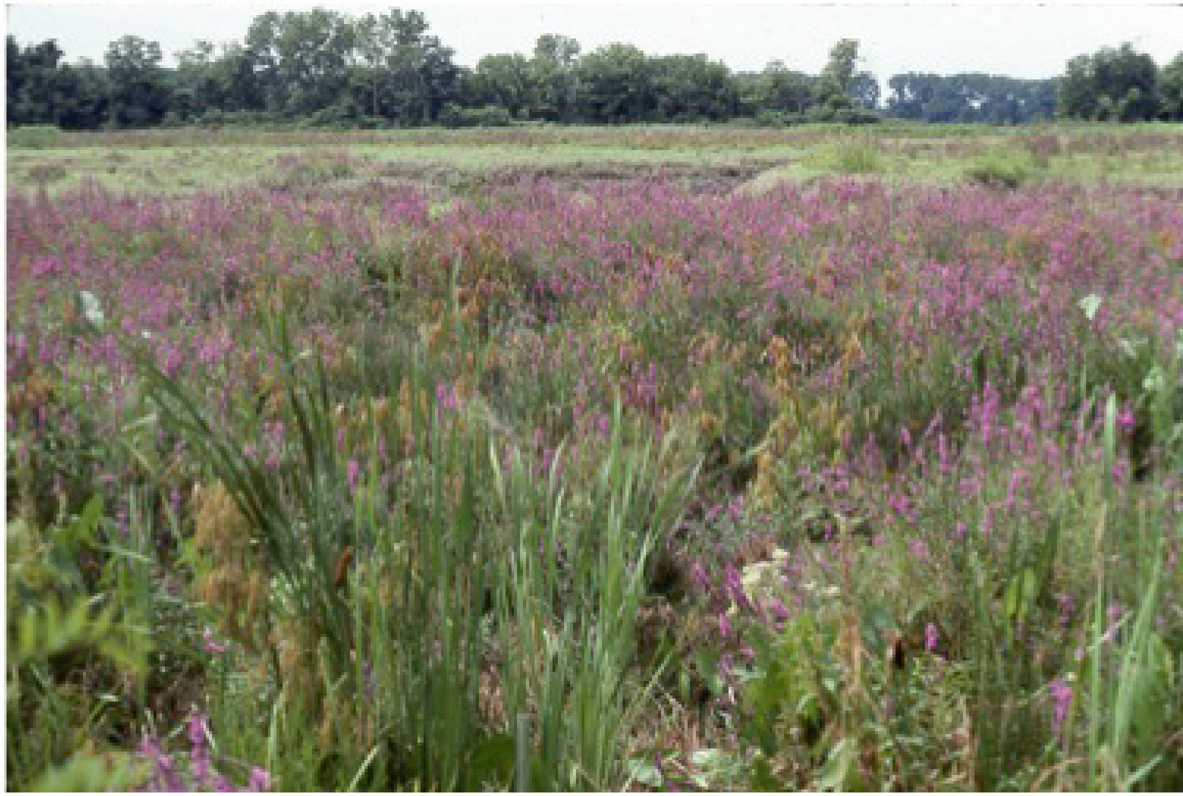

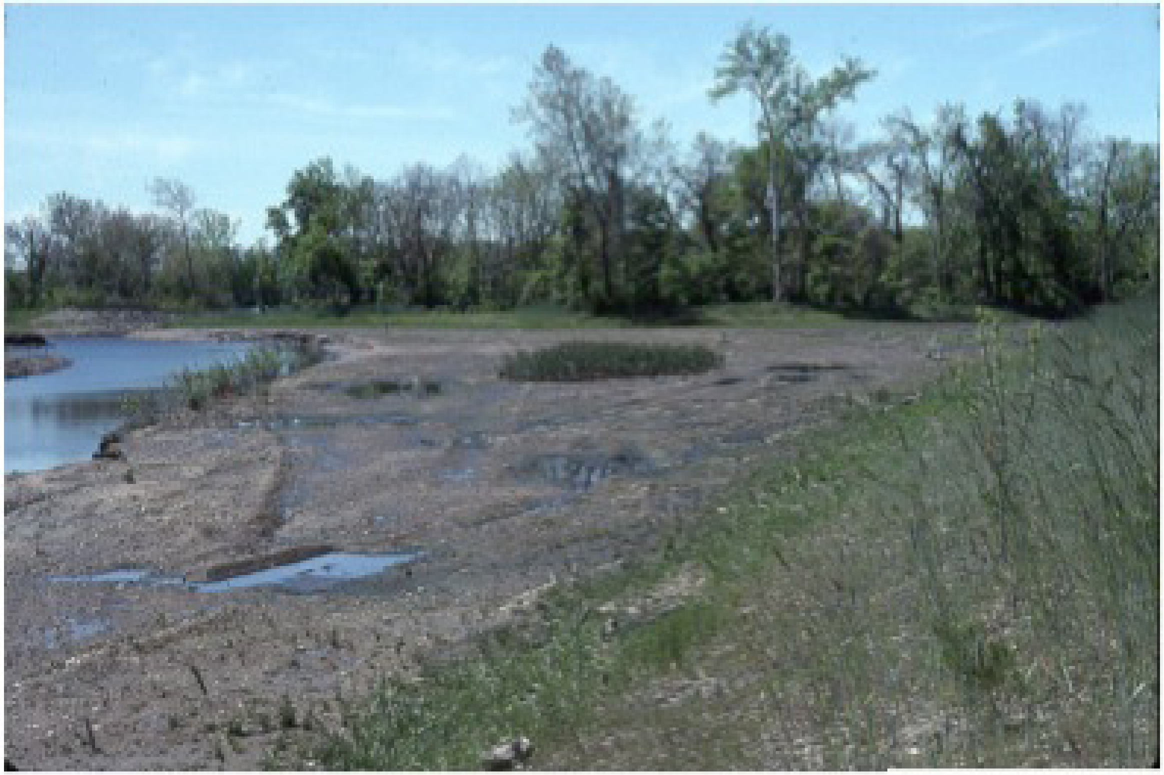

6 May '95

North Marsh

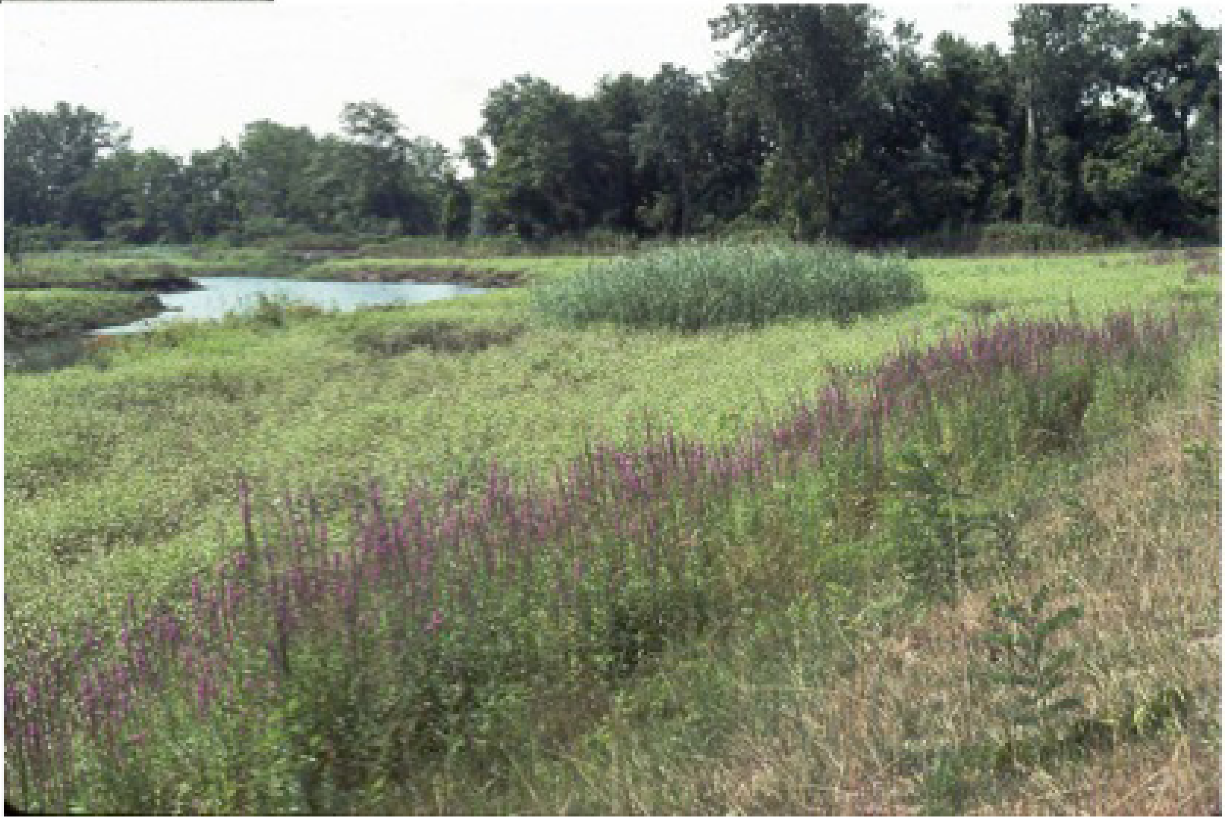

15 Aug. '95

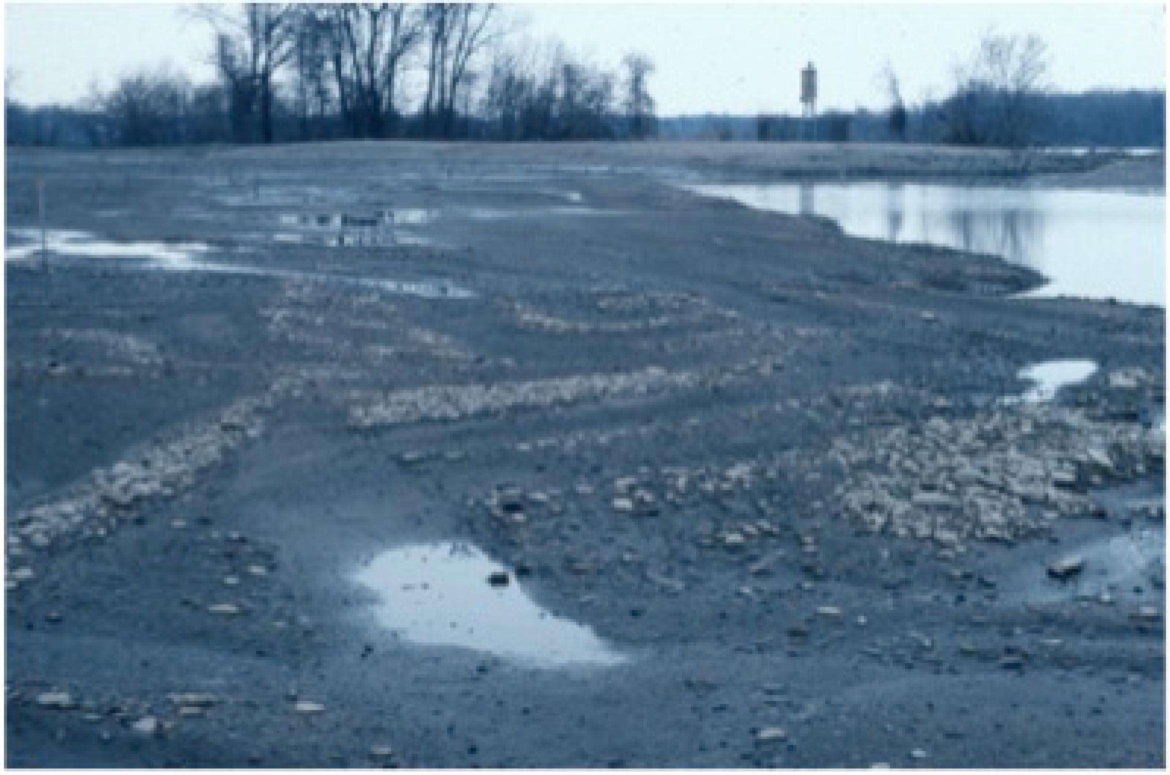

6 May '95

South Marsh

Late June '95

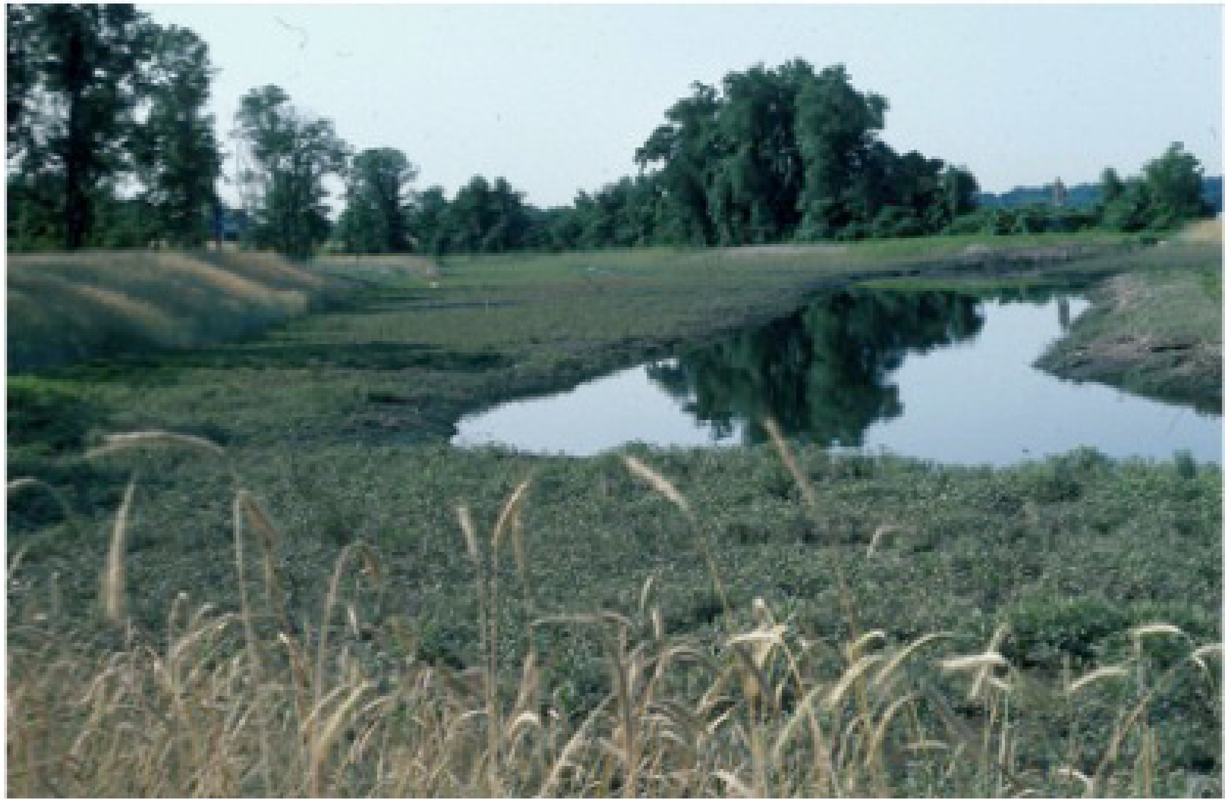

South Marsh: By Nov. 2002 the marsh vegetation was a mix of annuals and. perennials. Trees were well established along the upland edge.
Beyond the navigation beacon is the Delaware River.

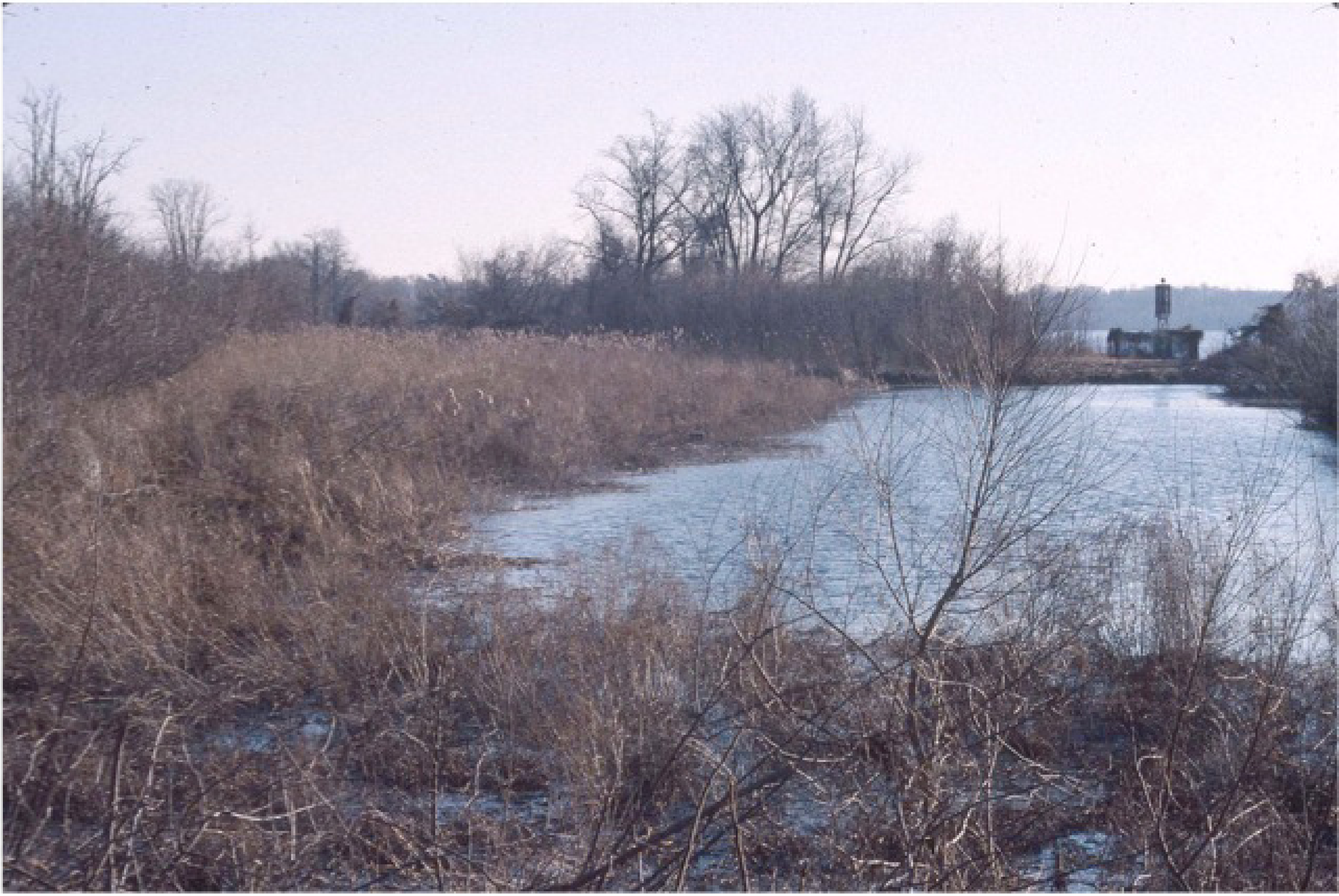

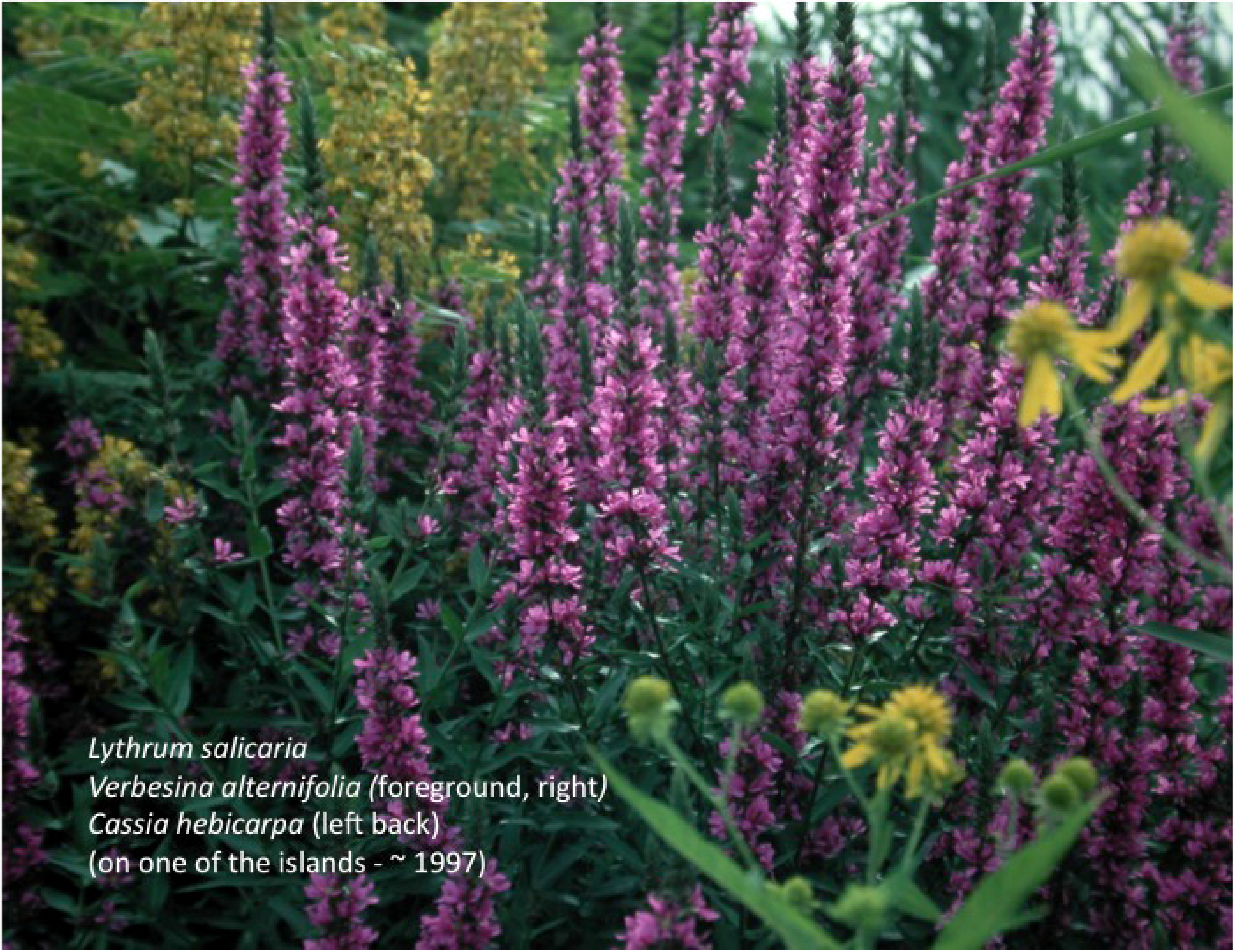
Lythrum salicaria
*Verbesina alternifolia (*foreground, right*)*
Cassia hebicarpa (left back)
(on one of the islands - ~ 1997)

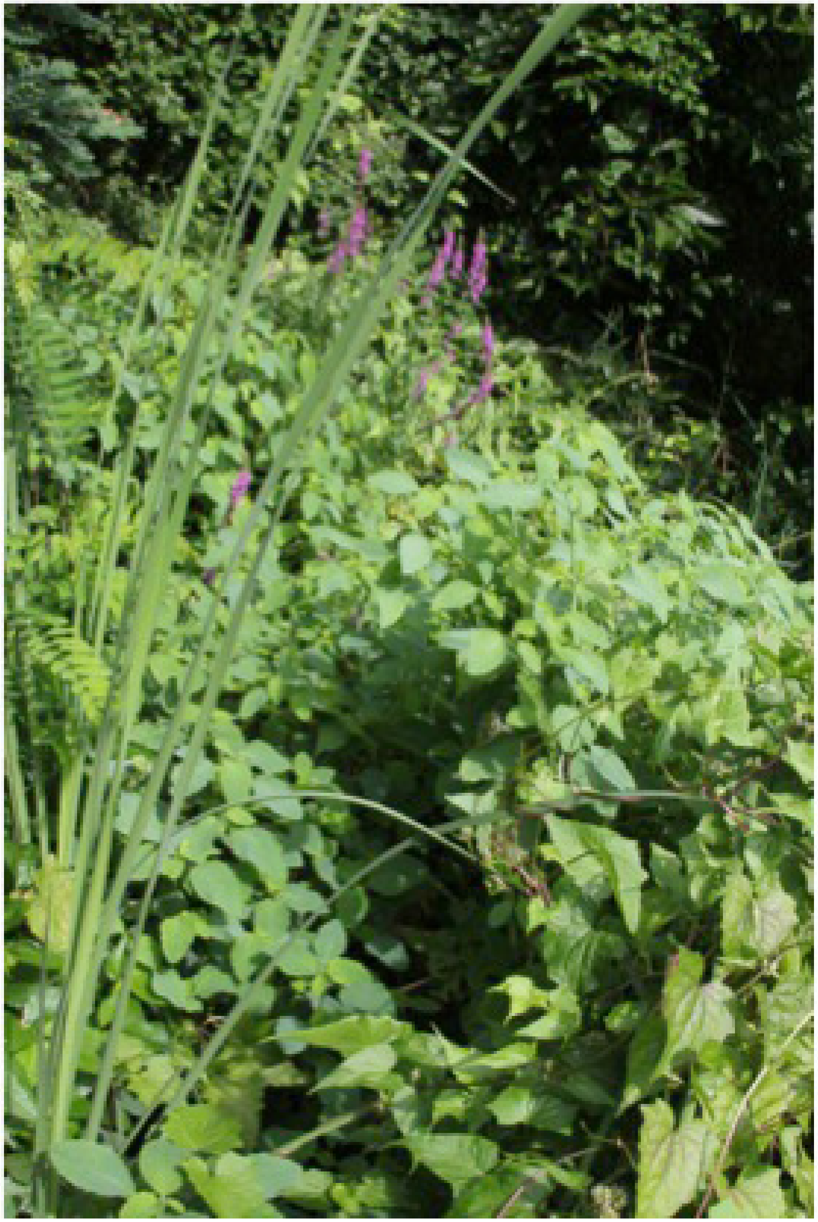

North Marsh (13 Aug. 2011)

Channel edge – *Typha angustifolia, Polygonum puctatum, Nuphar lutea; Phragmites australis*

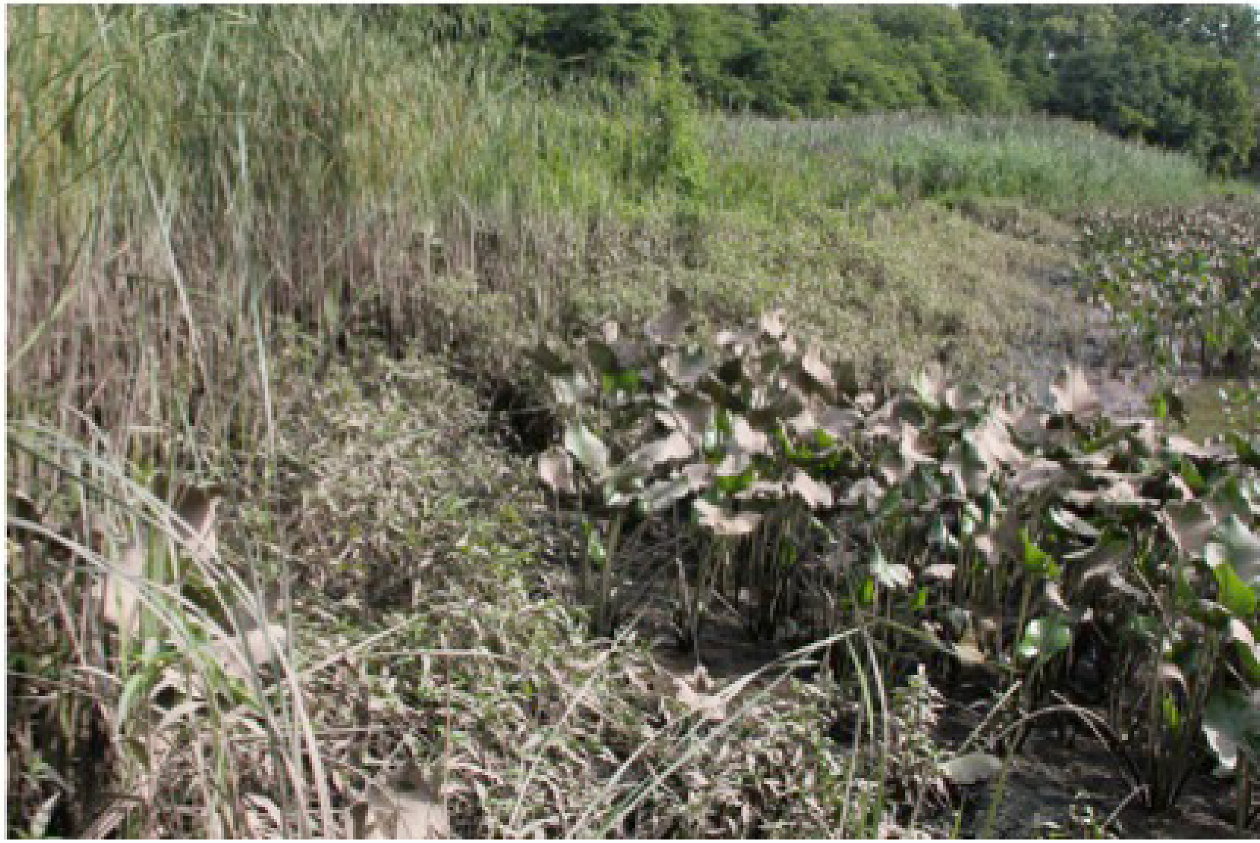

Upland edge –
Impatiens capensis, Typha sp., Mikania scandens, Lythrum salicaria (flowering)

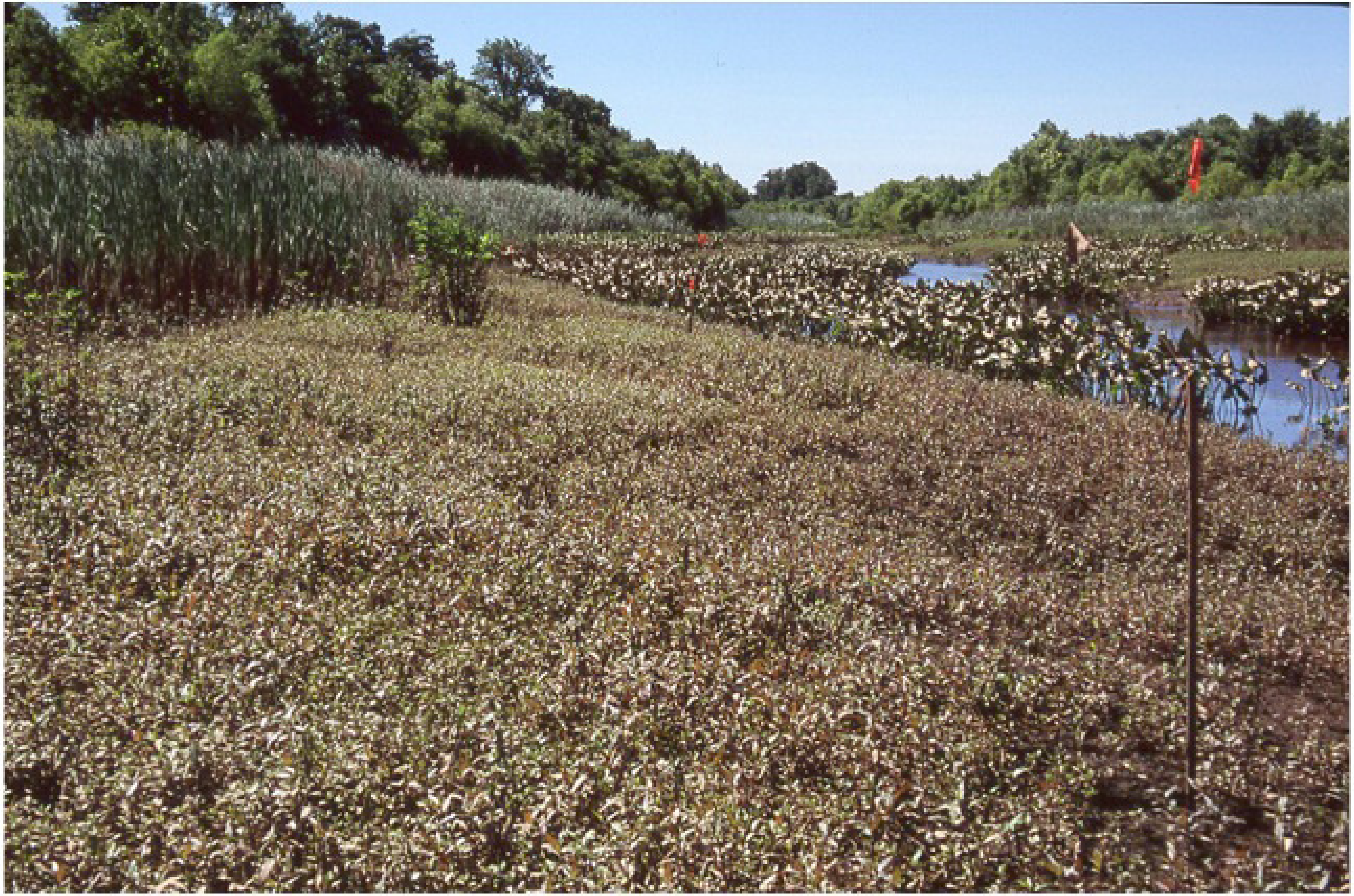

East Marsh - June 2004 (facing south)
Nuphar lutea in channel; *Polygonum punctatum* and others; *Typha latifolia* (left)
Phragmites australis beyond *Typha*.

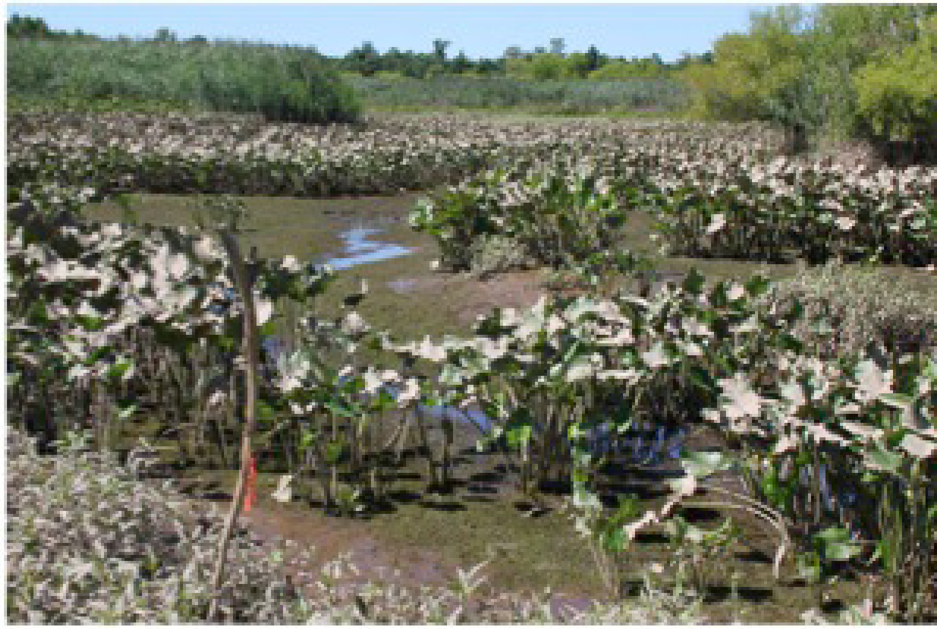

East Marsh channel –
facing west , *Nuphar lutea*

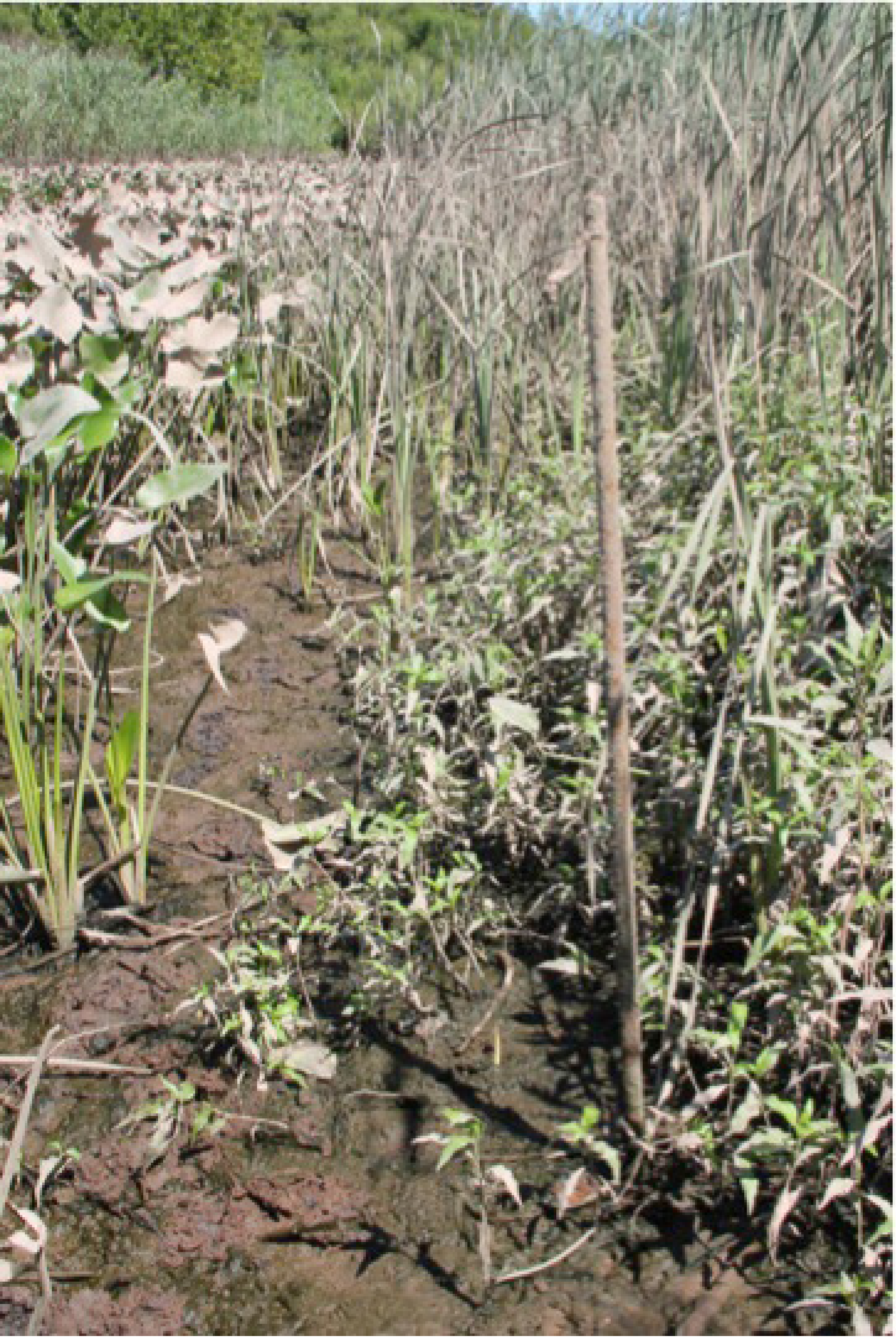

East Marsh (13 Aug. 2011) – channel edge
Nuphar lutea (left), *Polygonum punctatum*,
Typha angustifolia.

South Marsh (SM) in 2011. Note the red flagging on a transect marker in *Nuphar lutea*. Grass is *Zizania aquatica*.

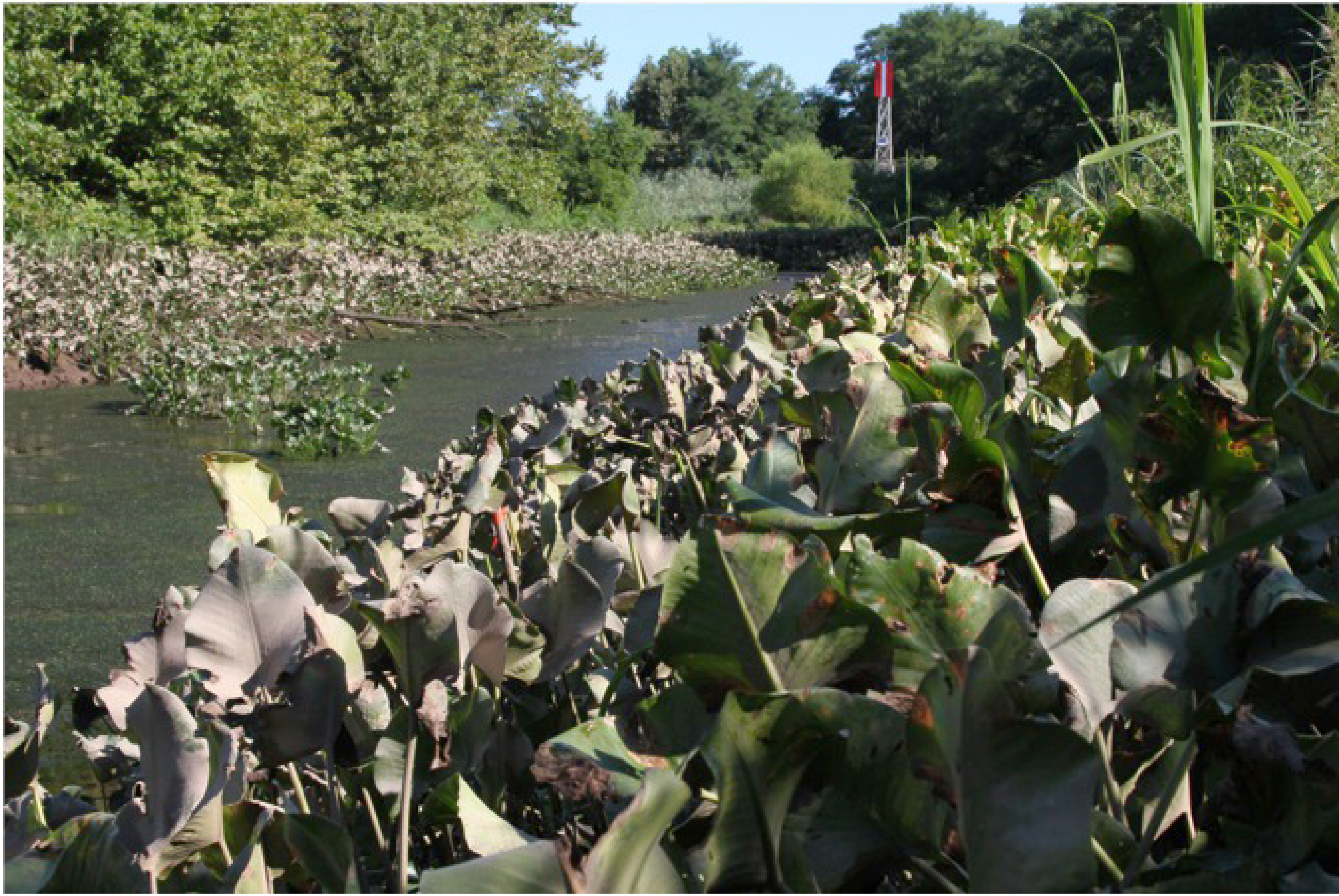

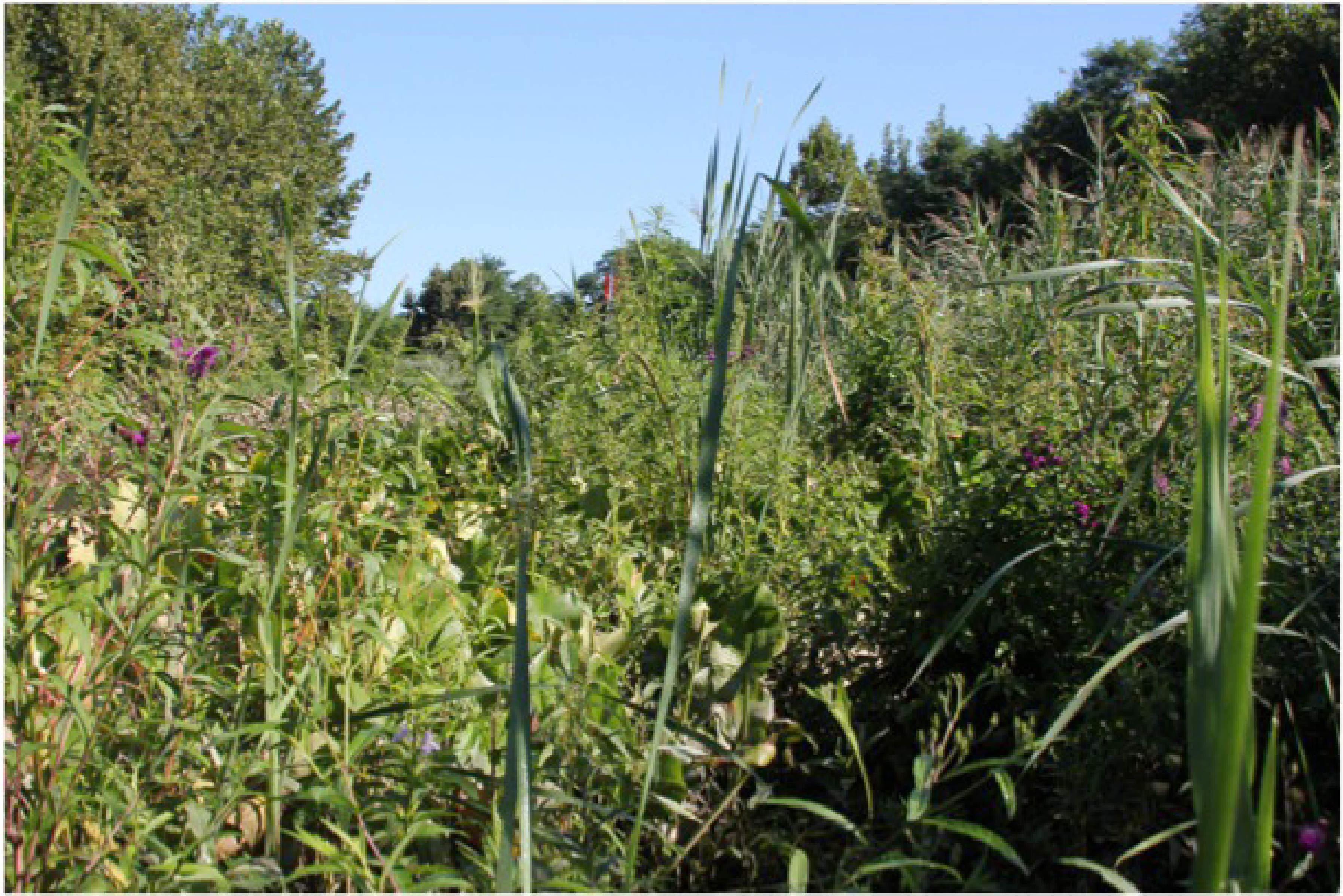

South Marsh (SM) (Aug 13, 2011) –channel edge, vegetation is a mix of species, including *Amaranthus cannabinus, Mimulus alatus, Nuphar lutea, Poyygonum punctatum, Symphyotrichum puniceum* (not flowering), *Typha latifolia, Zizania aquatica.* *Phragmites australis* (visible on the right) has not yet invaded the channel edge.

.

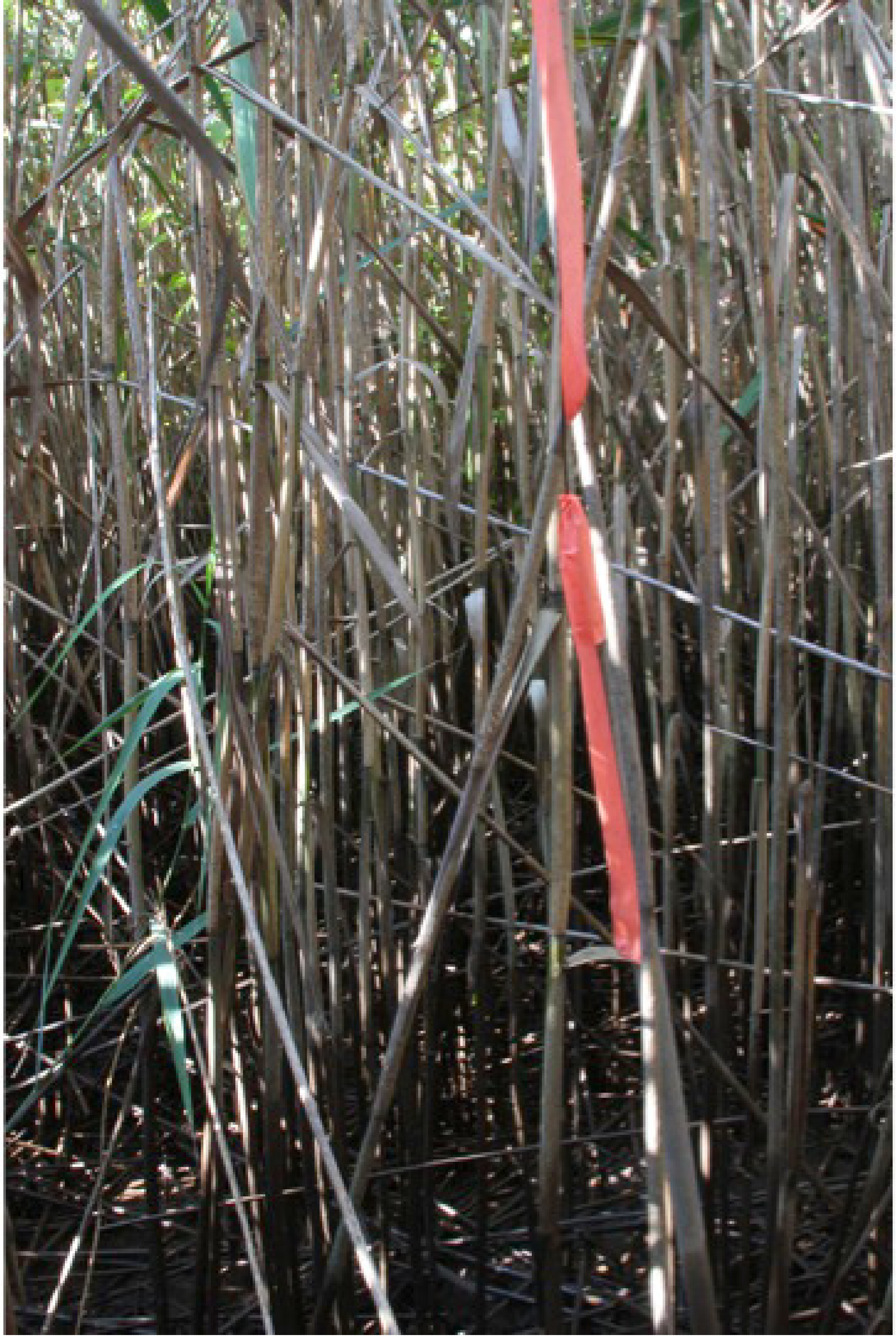

East Marsh (13 Aug. 2011)
Phragmites australis
patch.

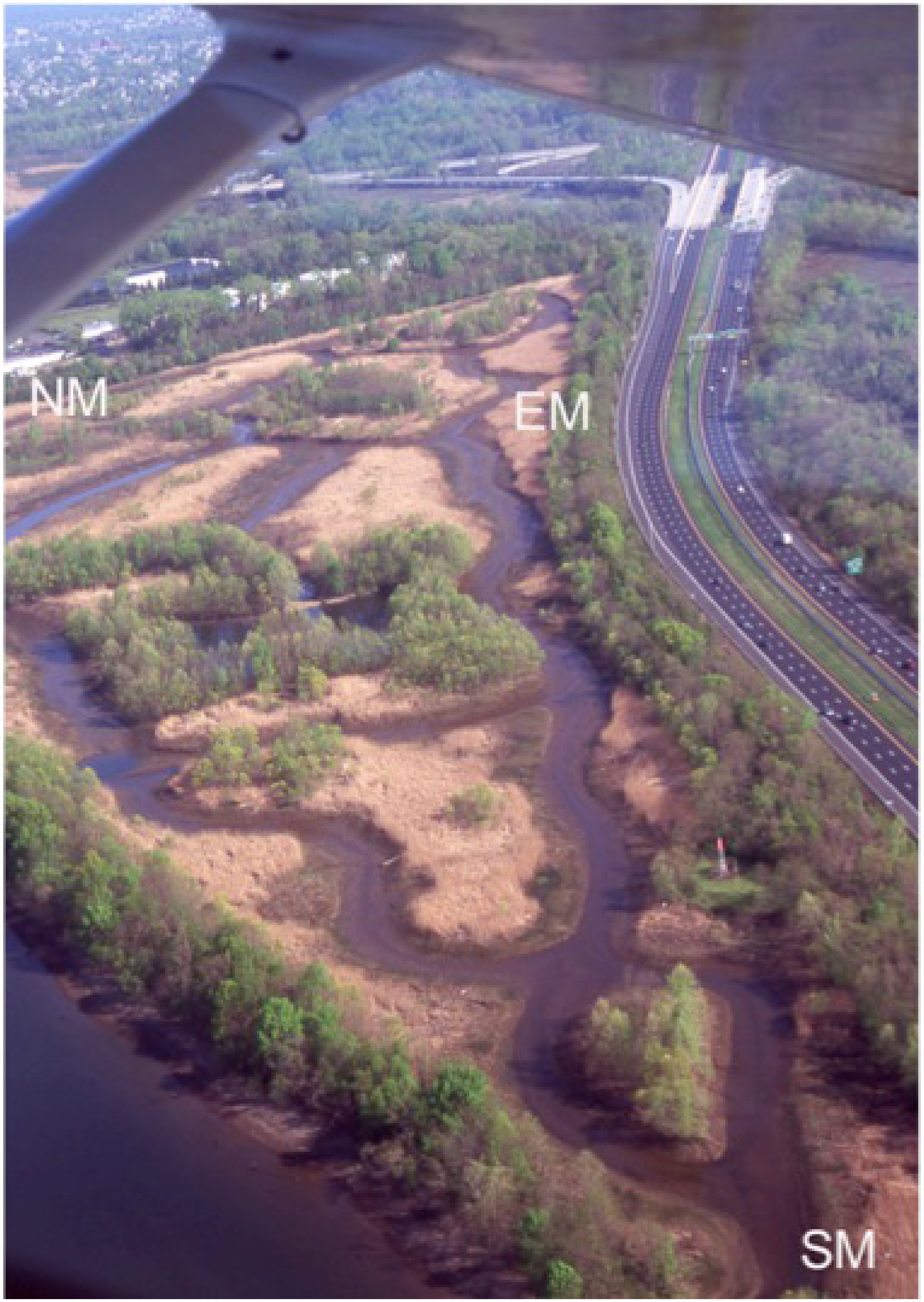

Duck Island – created wetland
Aerial –mid April 2010, facing North; I-295 is at right and to east is the Abbott Marshlands

Phragmites australis thatch from 2009 growth is light brown.

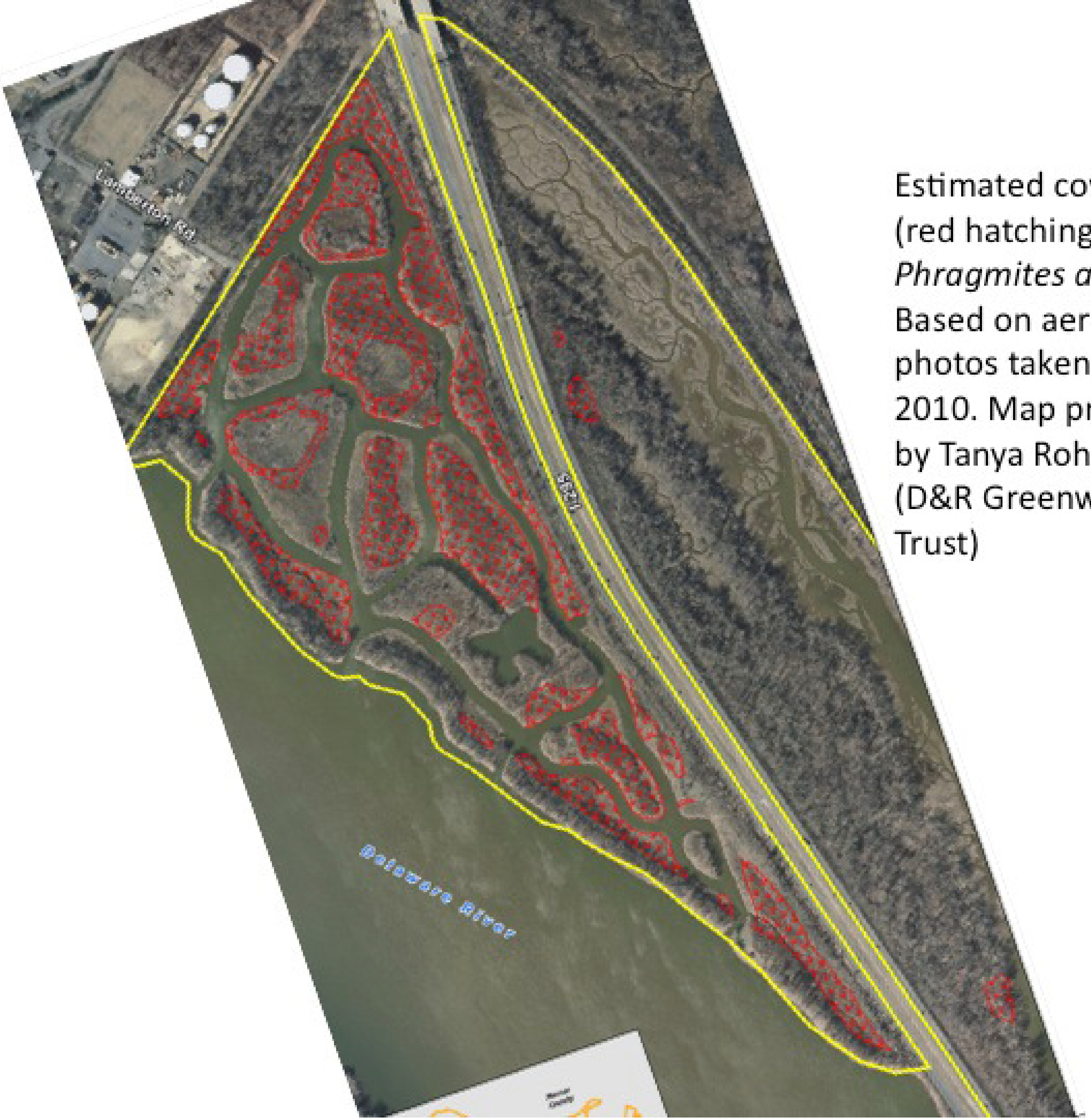

Estimated coverage (red hatching) of *Phragmites australis*. Based on aerial photos taken April 2010. Map prepared by Tanya Rohrbach (D&R Greenway Land Trust)

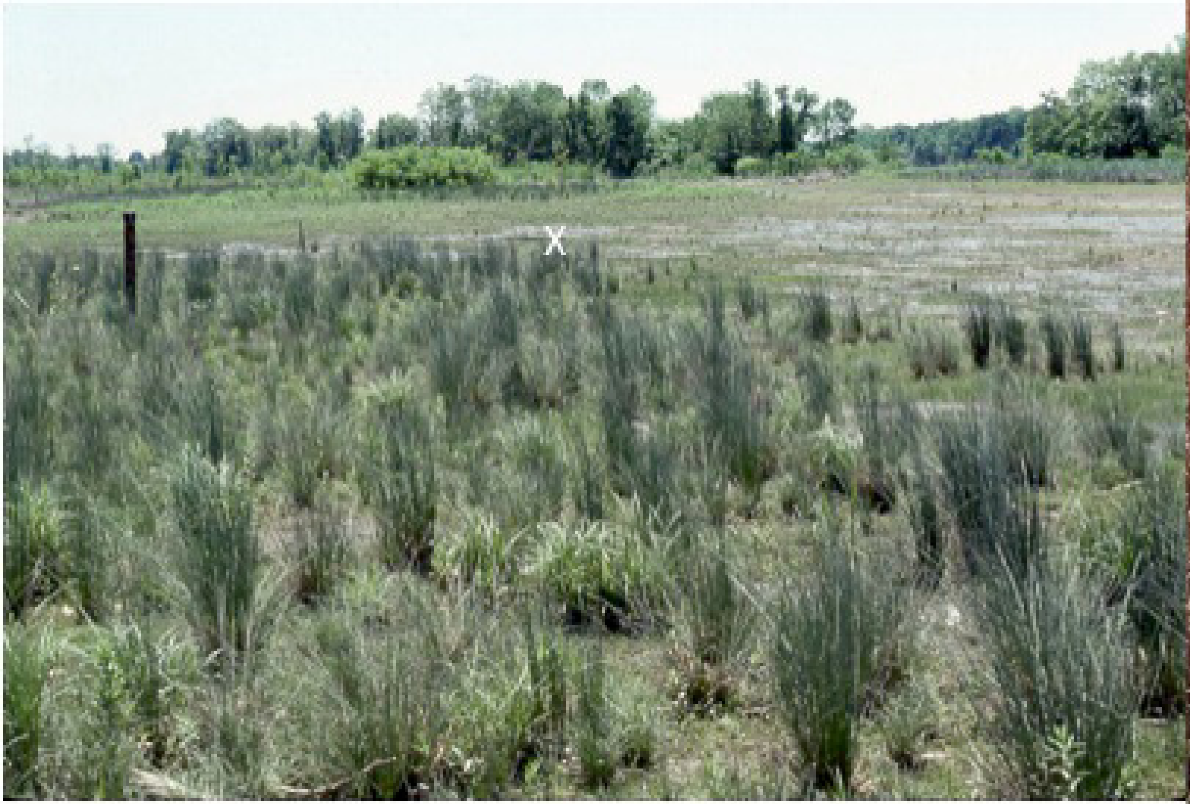

25 May 1996, looking south.
X= approximate location of
Oct. photo

Elantine Island

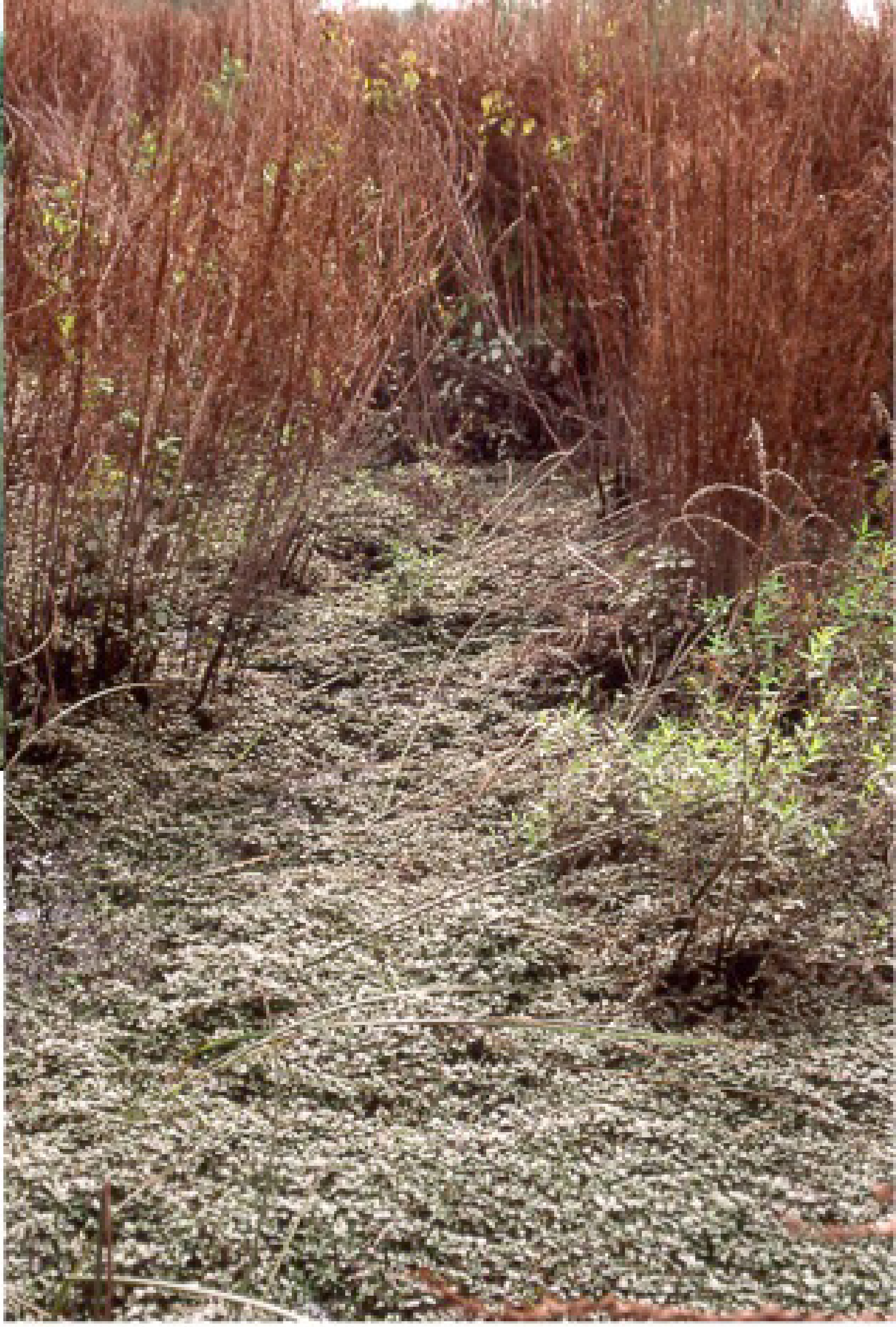

7 October 1999 – *Lythrum salicaria*
with mat of *Ludwigia palustris*
