# Supplementary material for: Dispersal potential of a tidal river and colonization of a created tidal freshwater marsh
Source: AoB Plants. 2012 Dec 21;5:pls050. doi: 10.1093/aobpla/pls050 (PMC4104633; doi:10.1093/aobpla/pls050)

## Slide 1
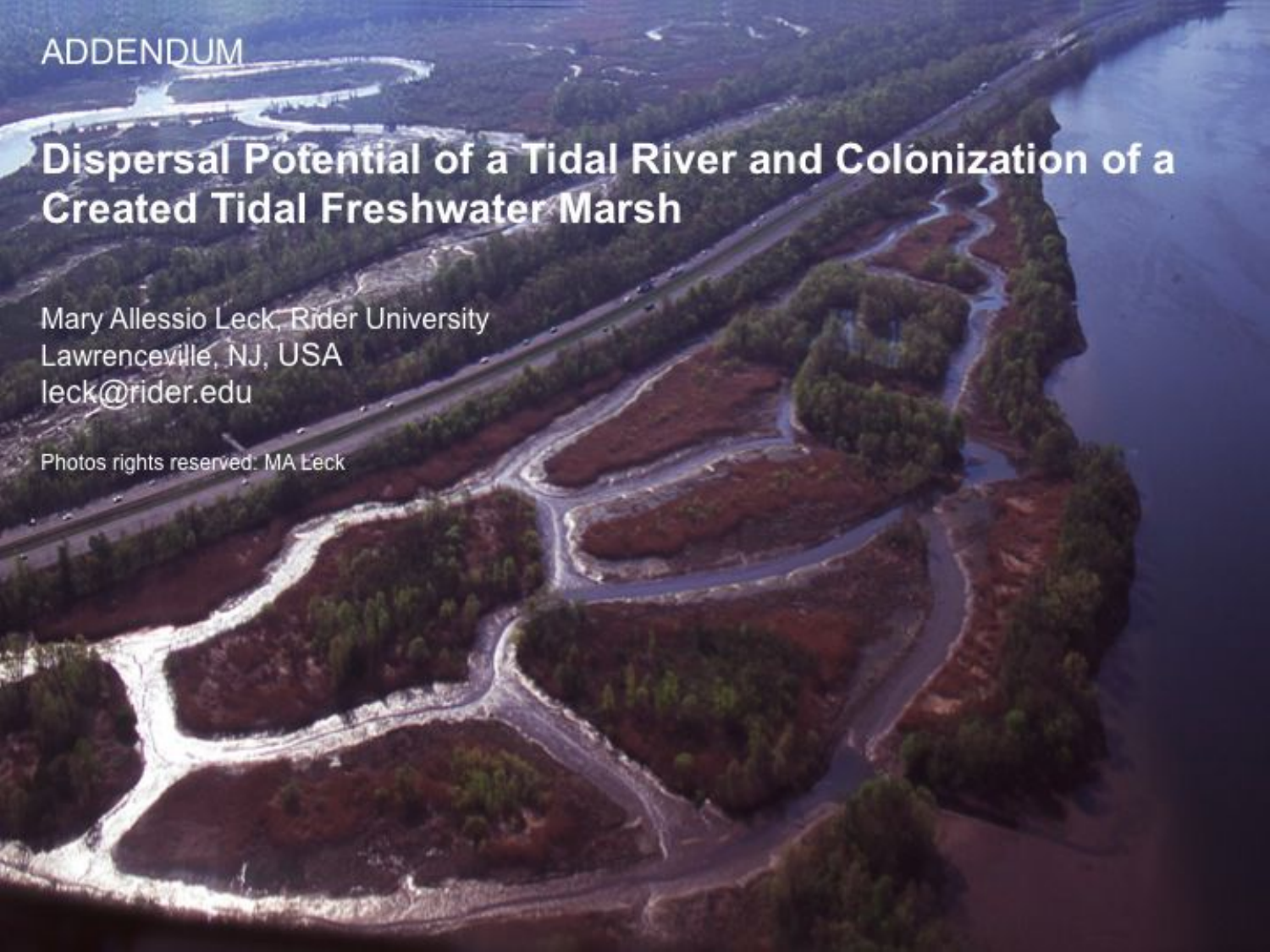

## Slide 2
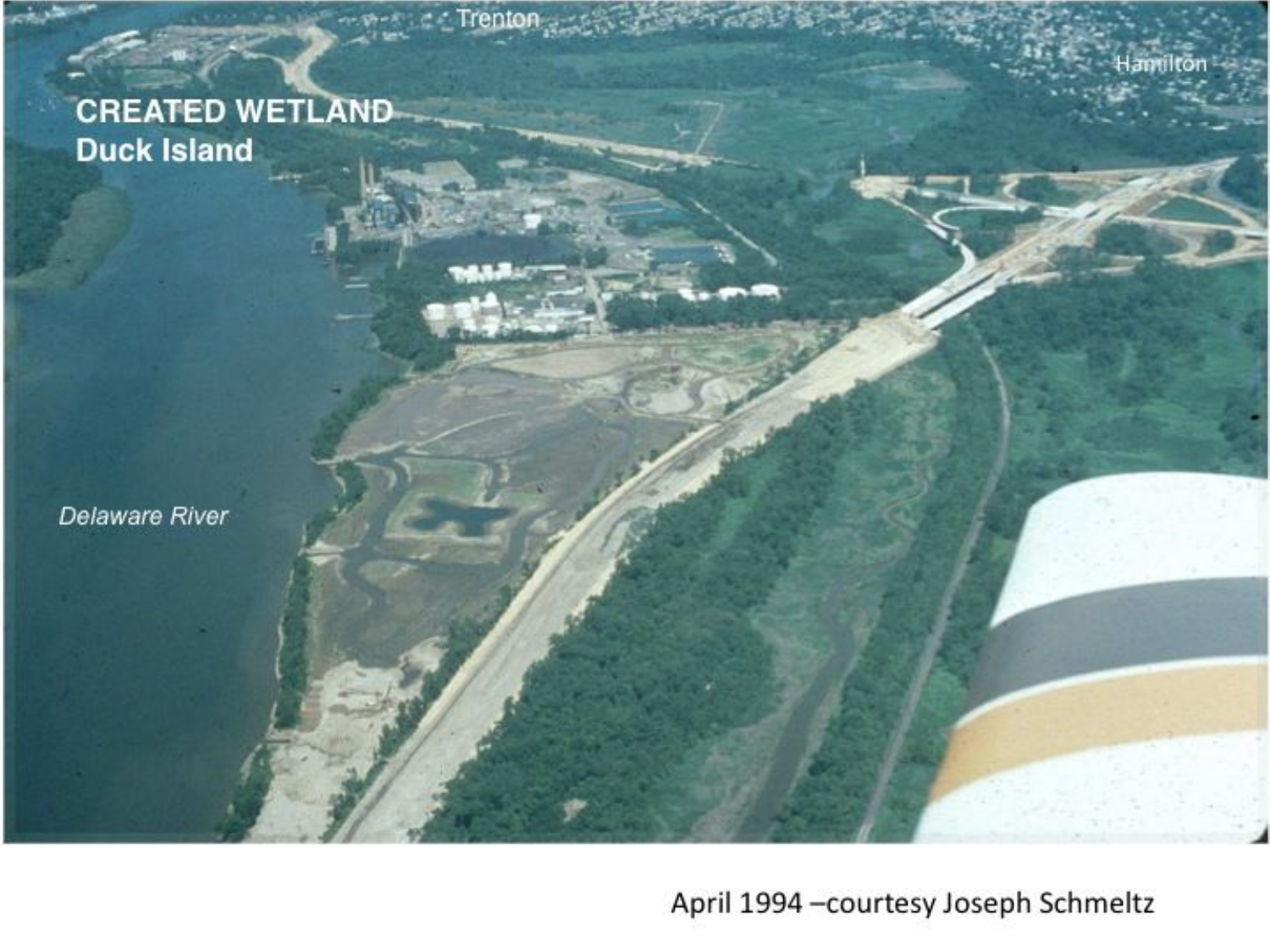

## Slide 3
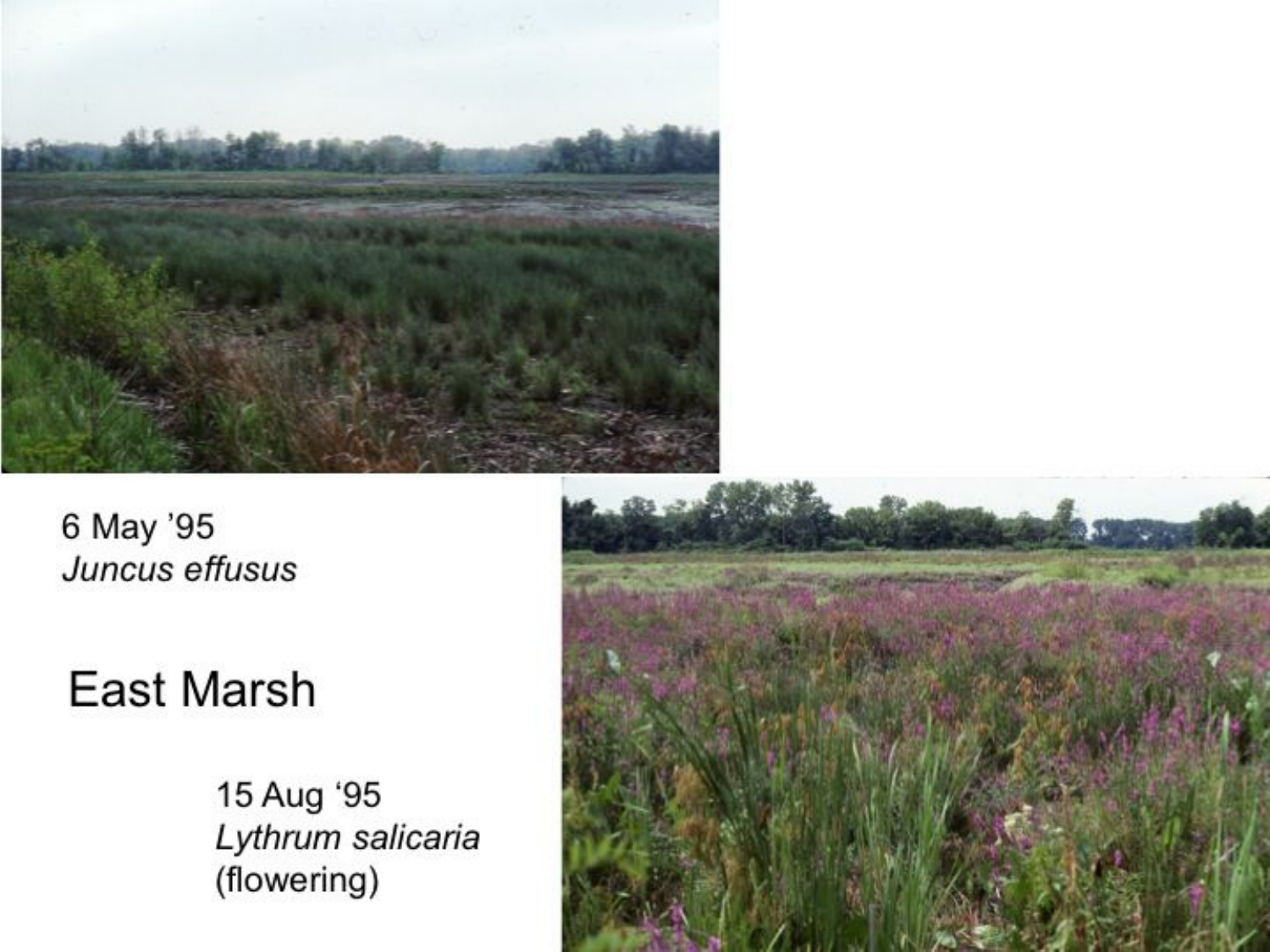

## Slide 4
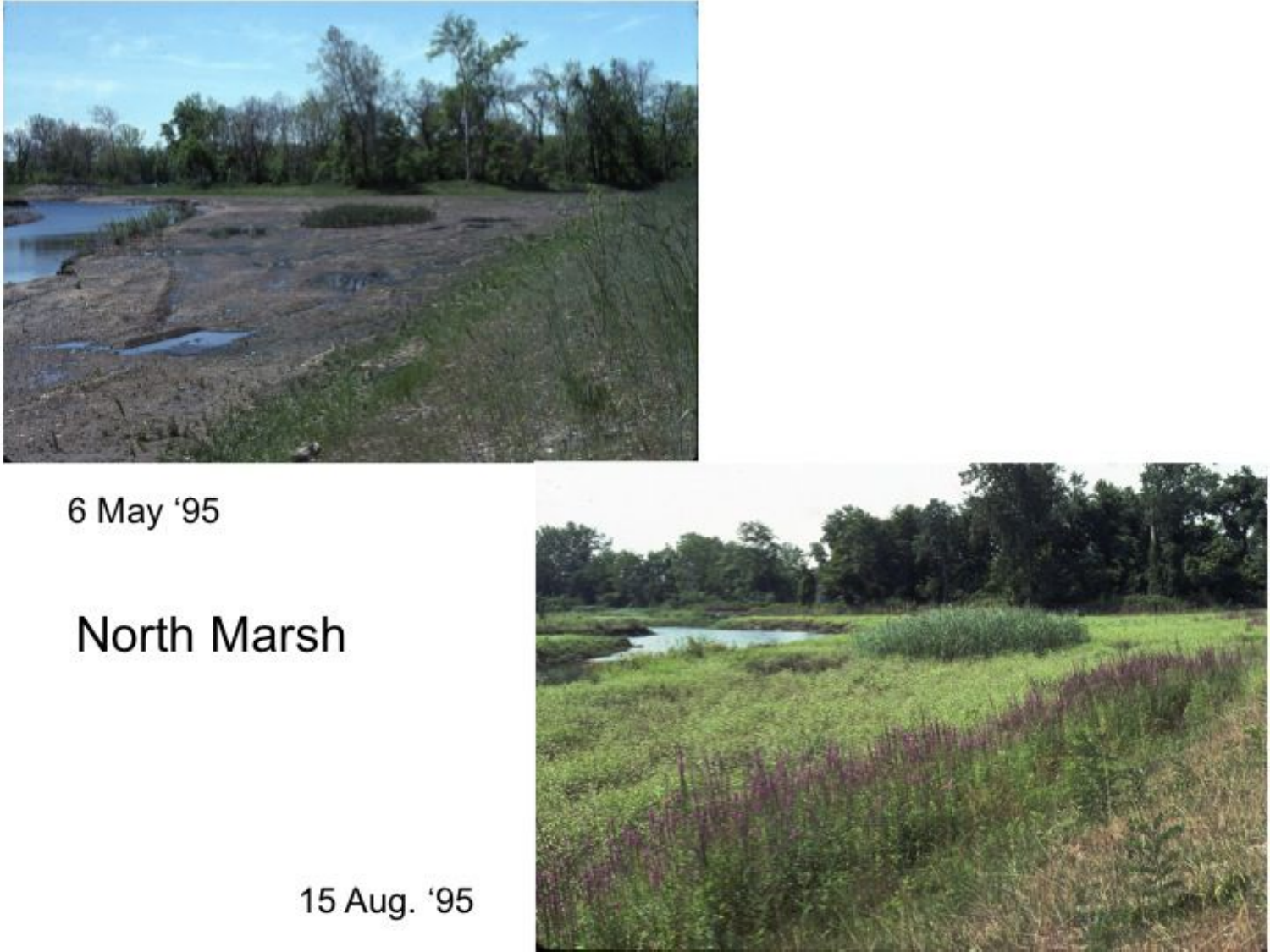

## Slide 5
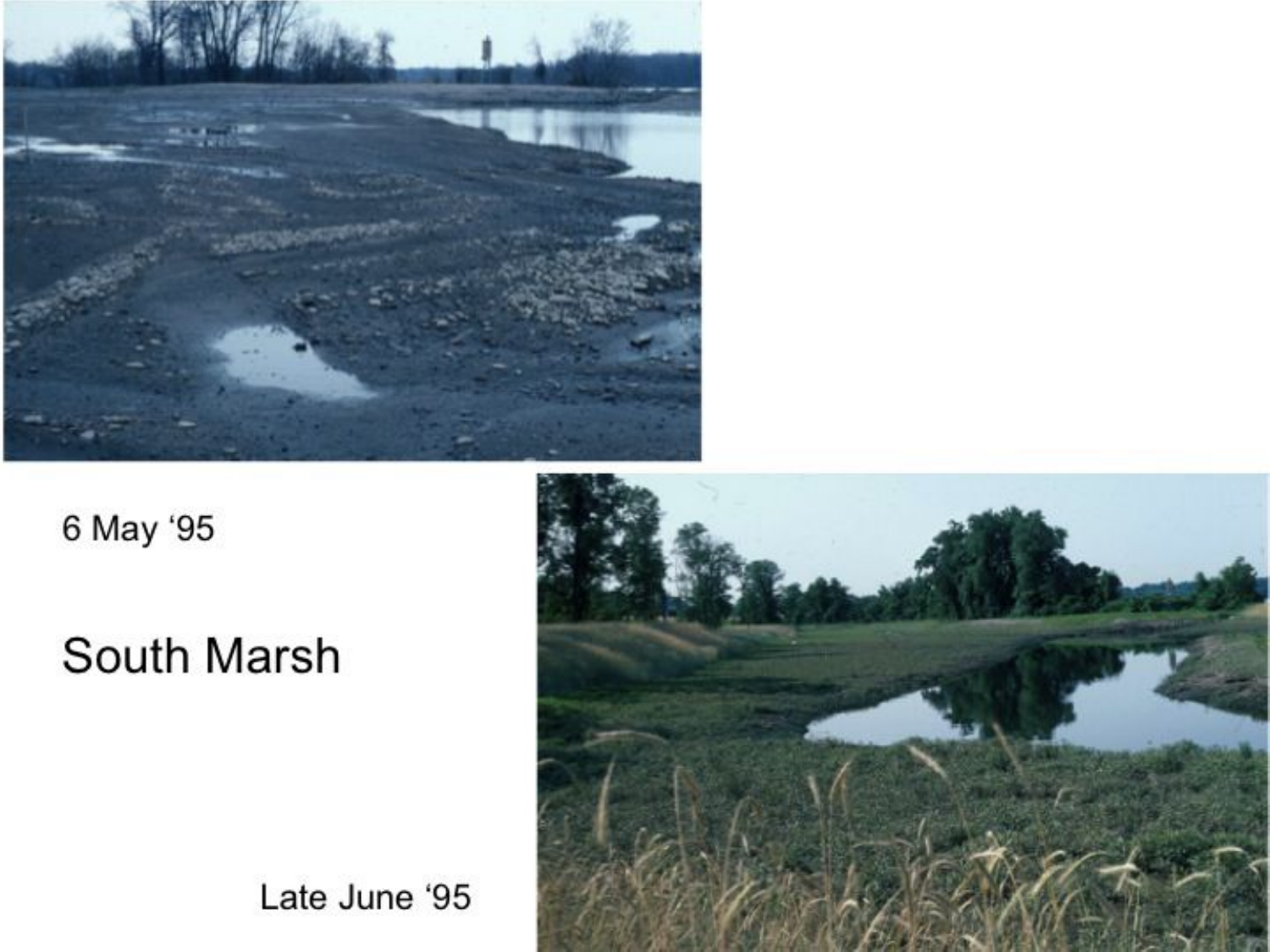

## Slide 6
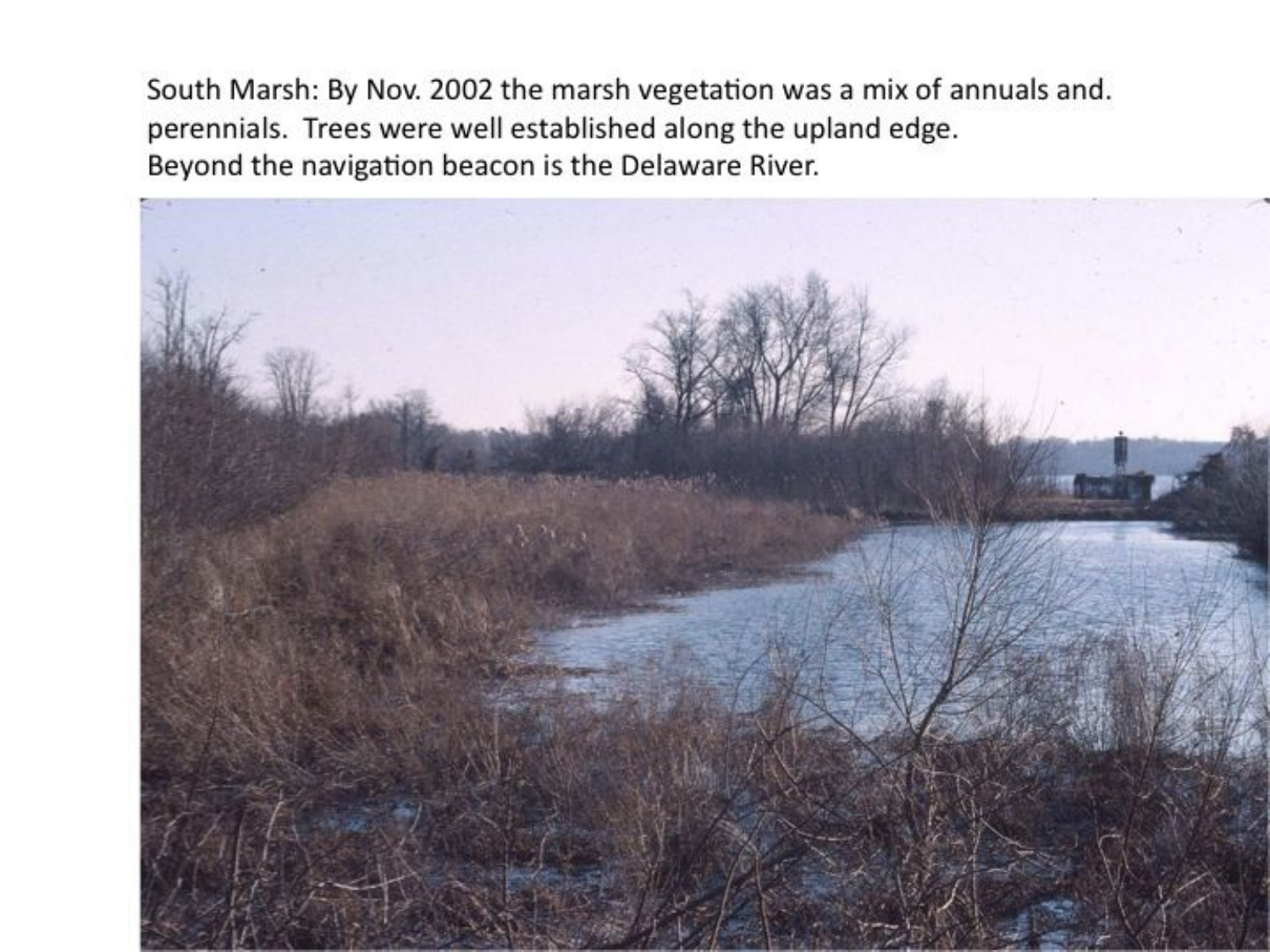

## Slide 7
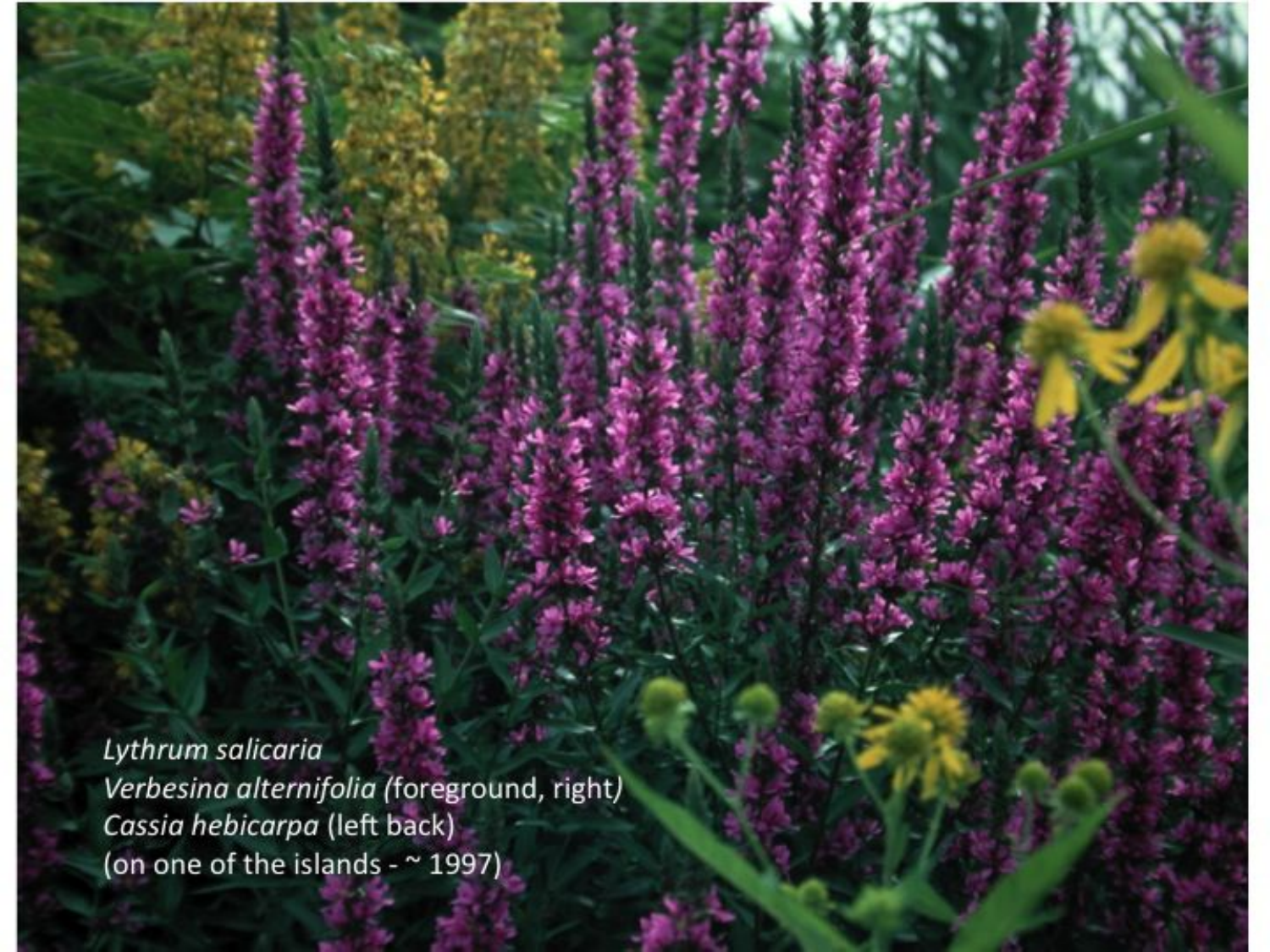

## Slide 8
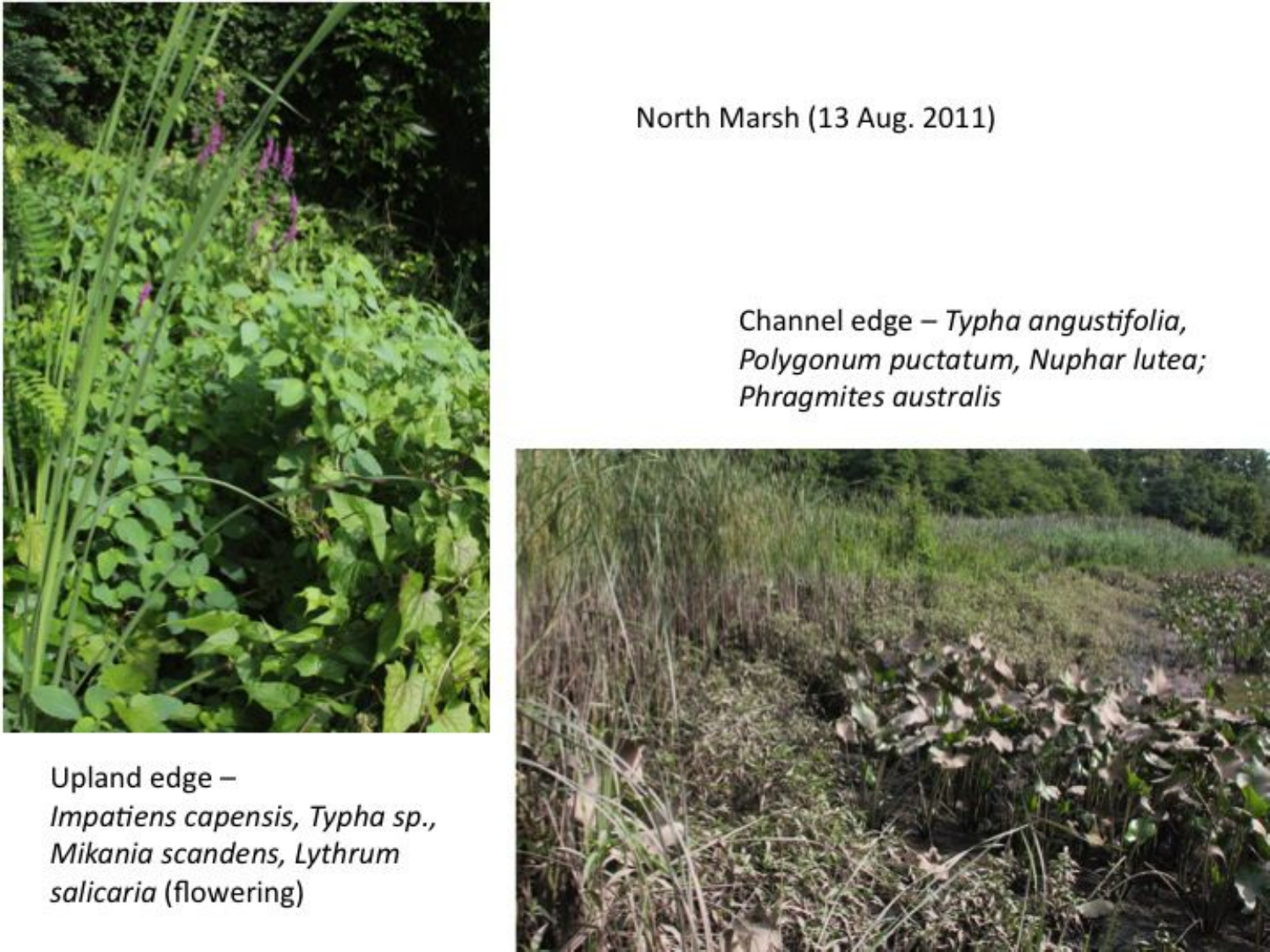

## Slide 9
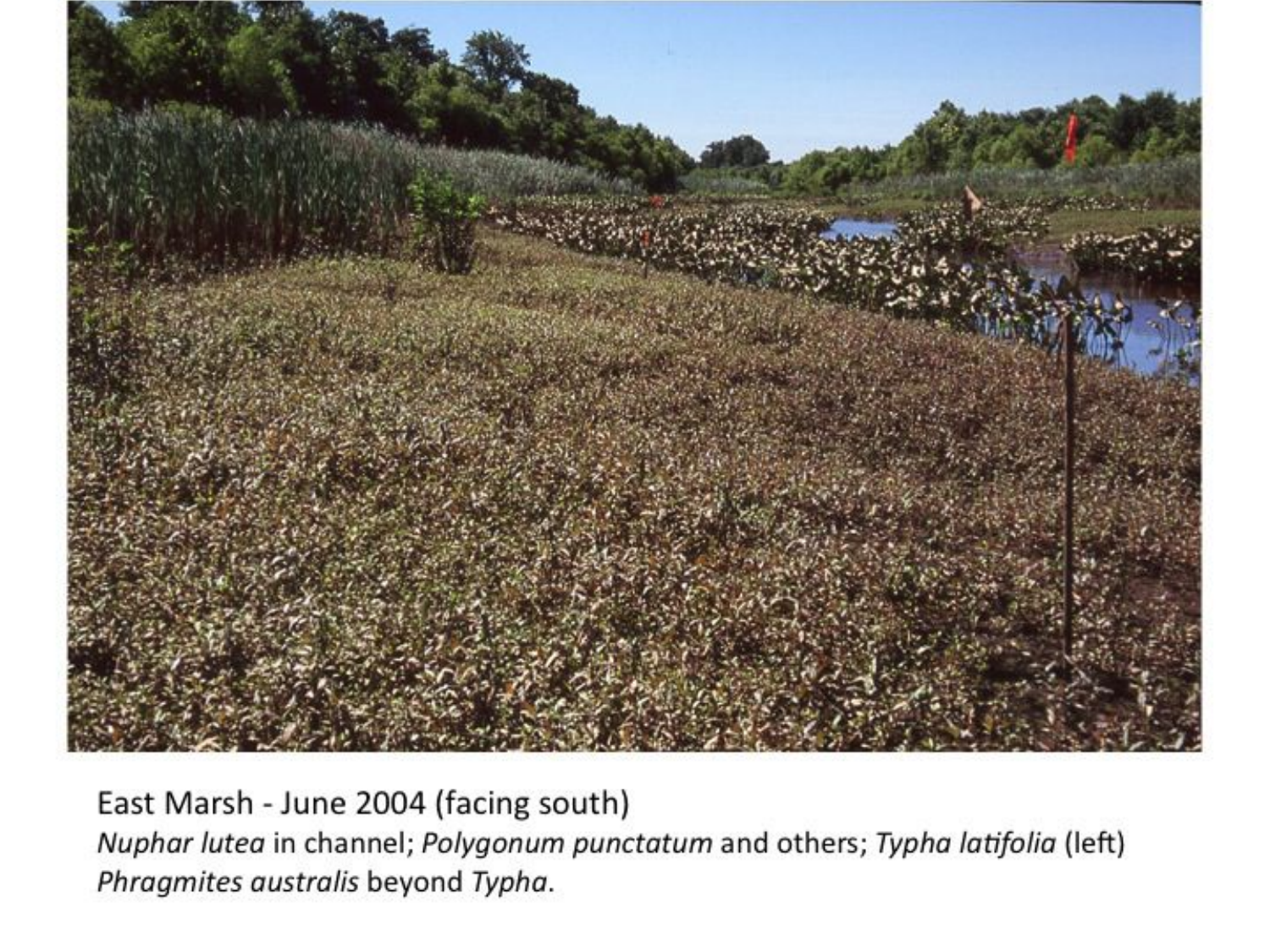

## Slide 10
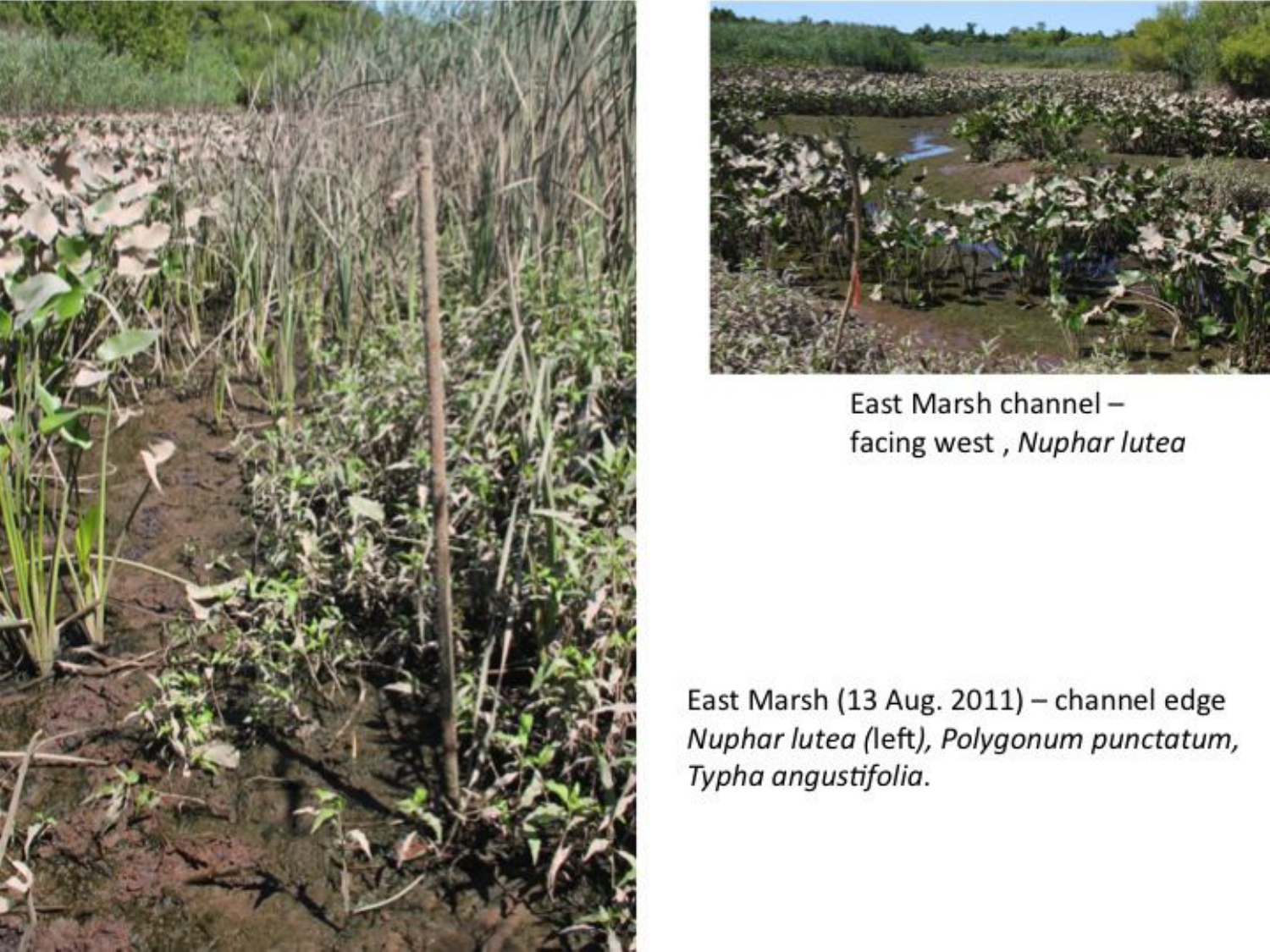

## Slide 11
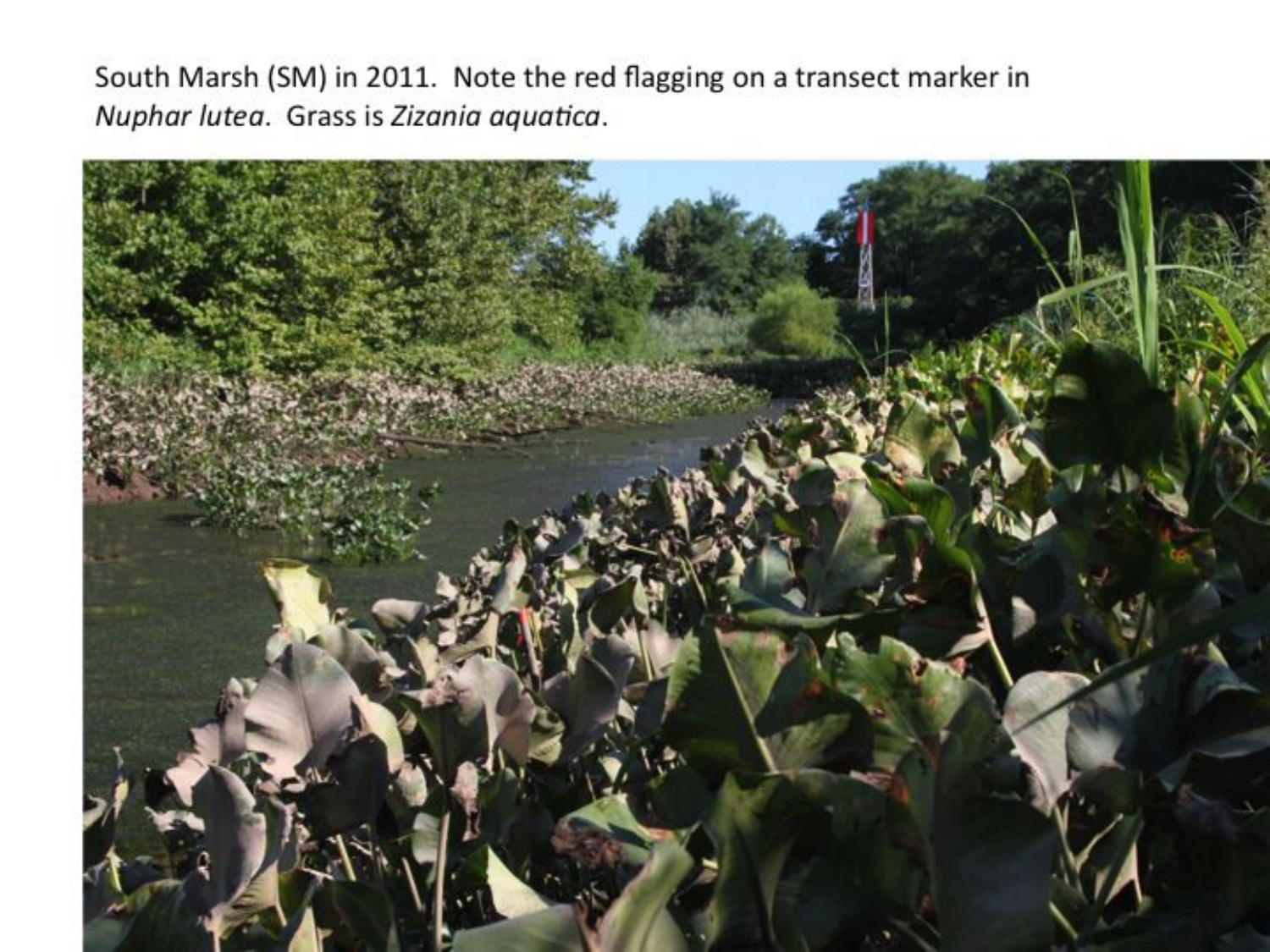

## Slide 12
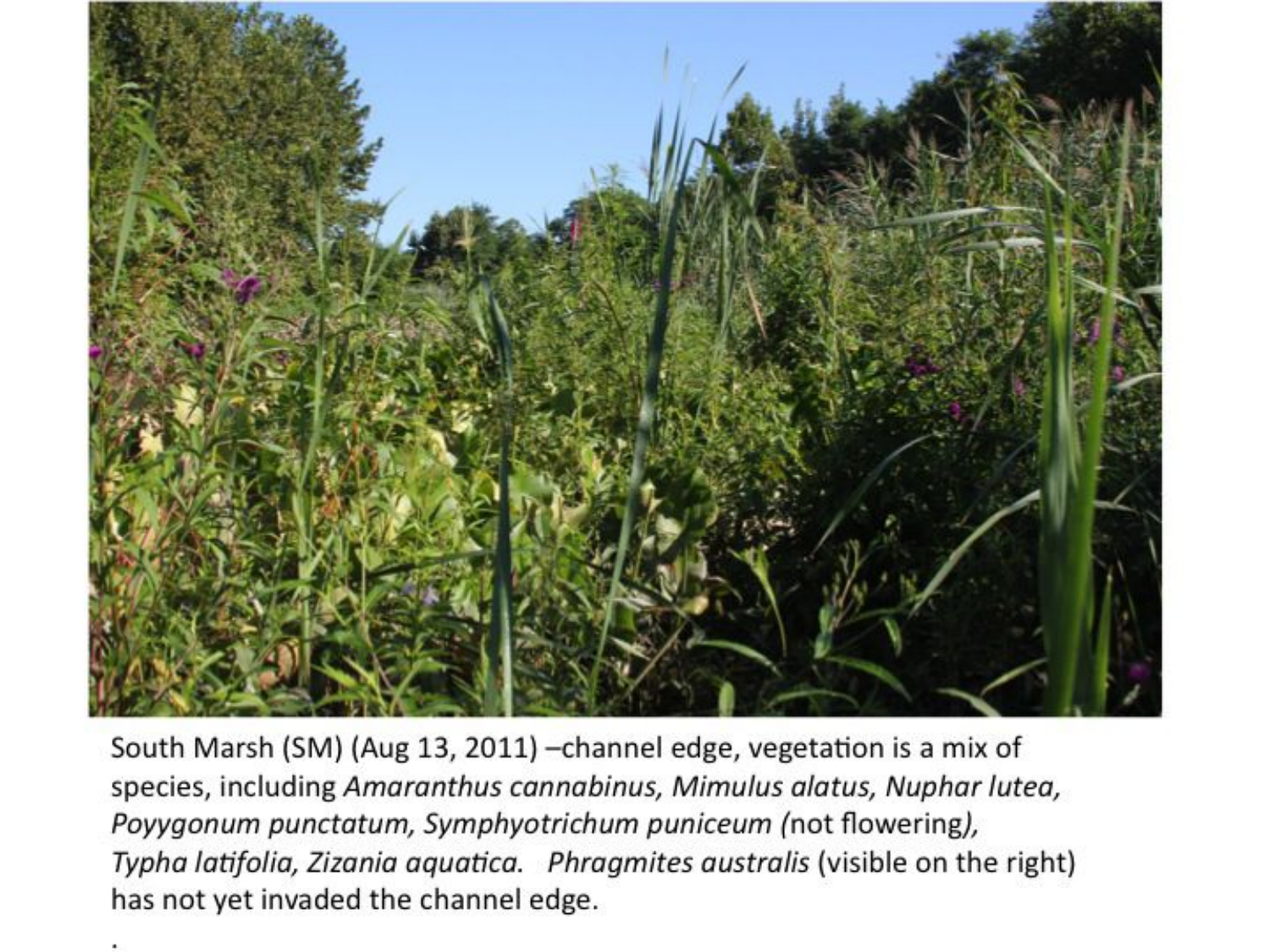

## Slide 13
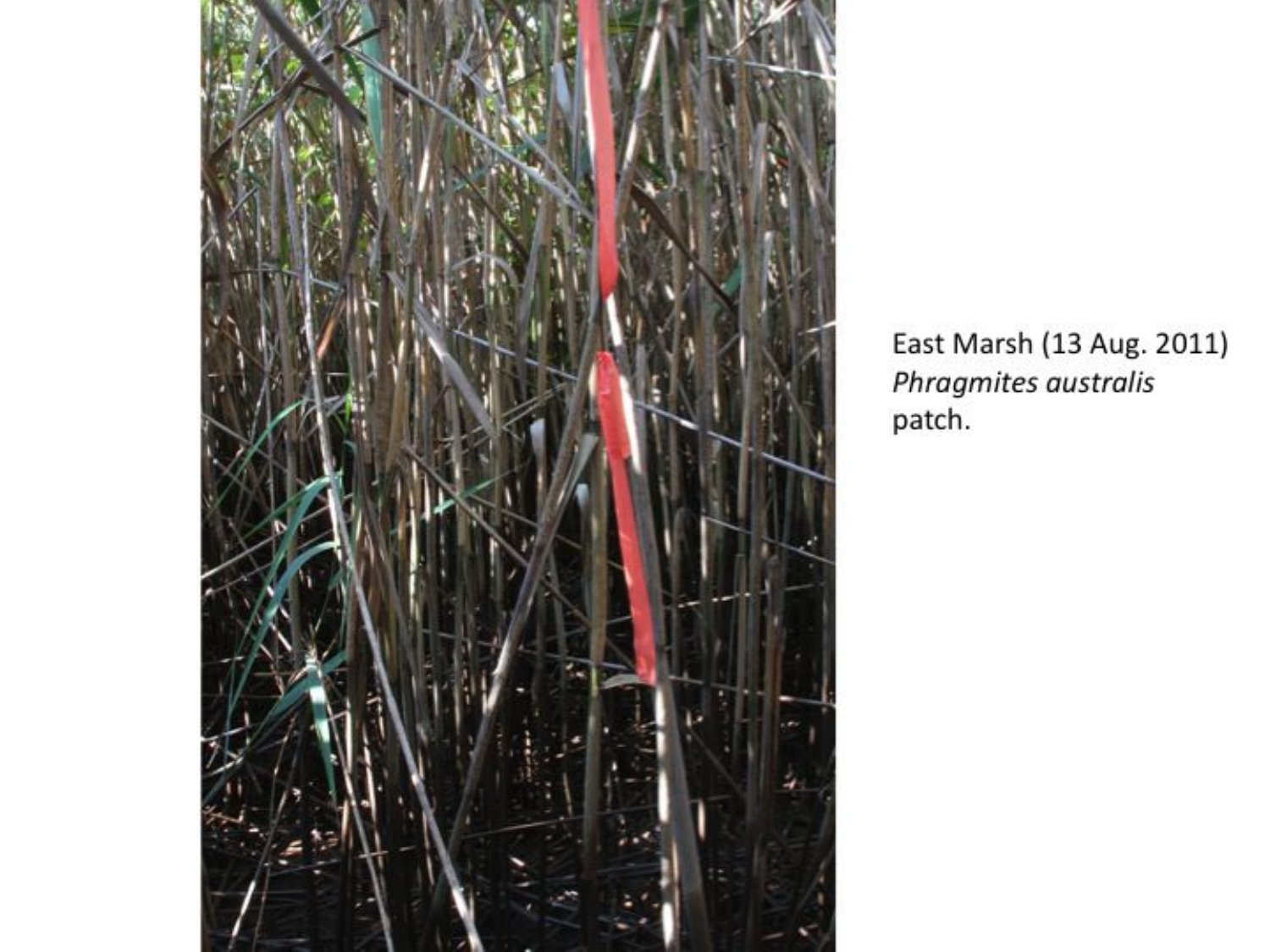

## Slide 14
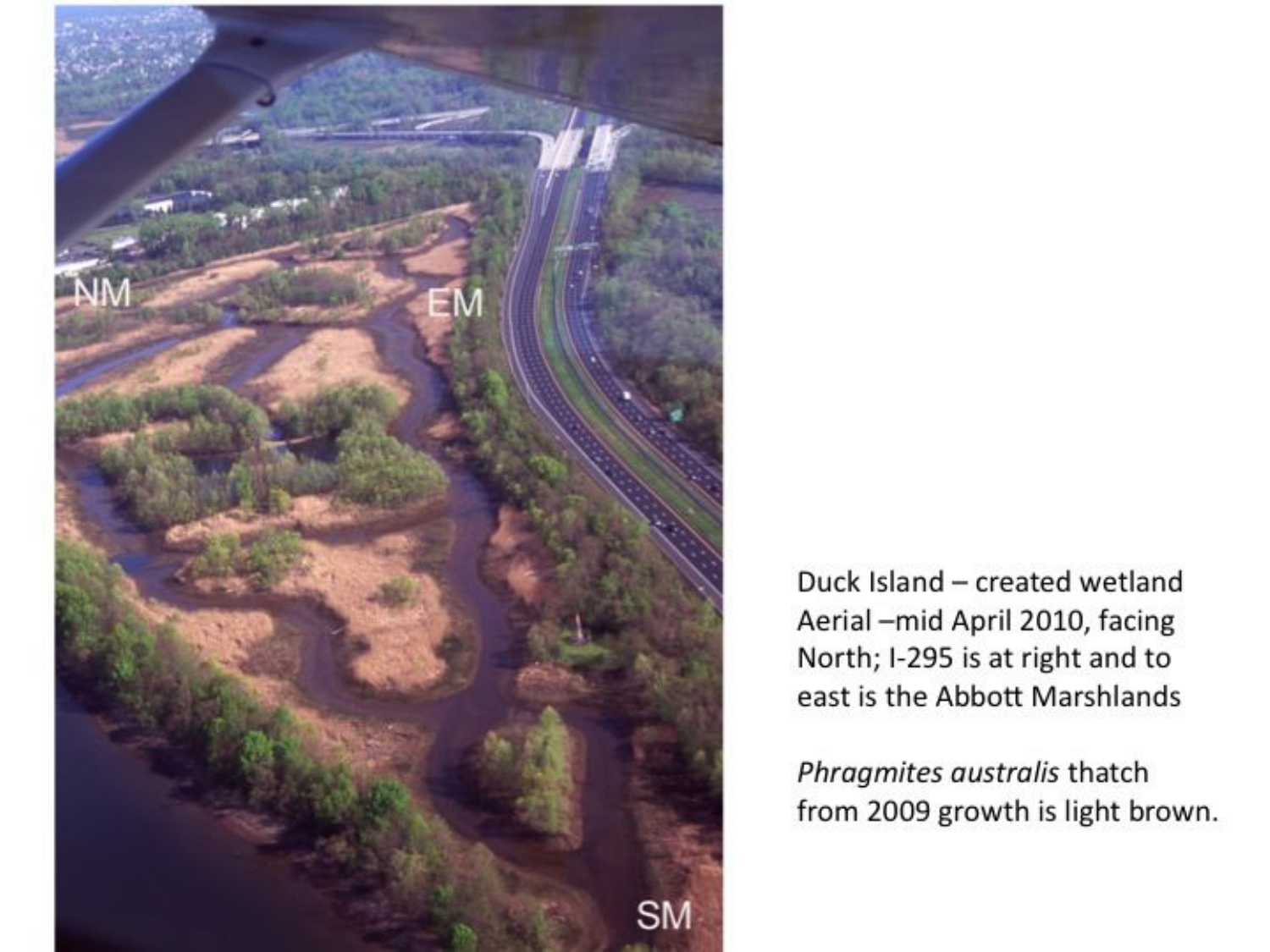

## Slide 15
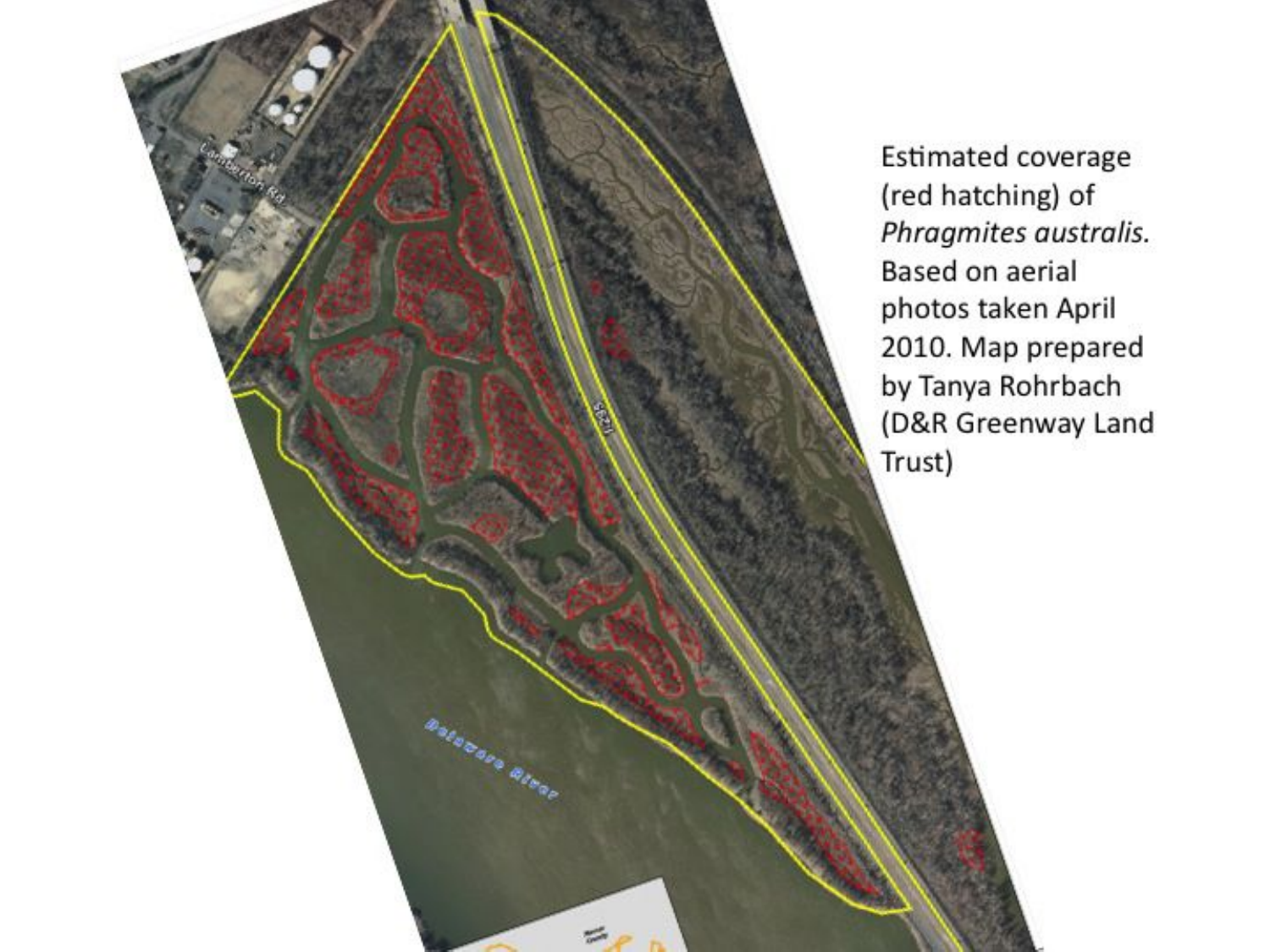

## Slide 16
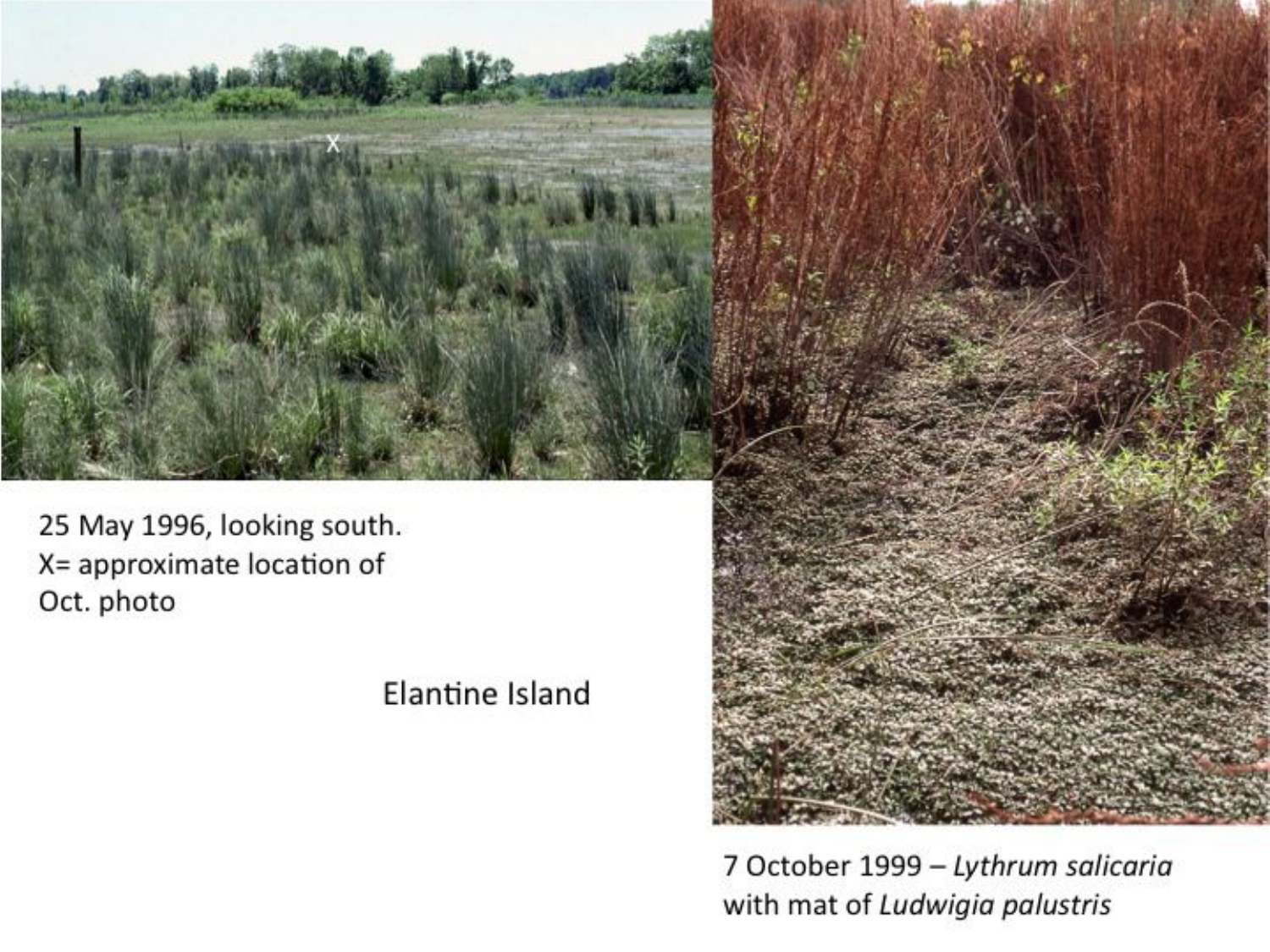

Supplement: Additional Information [file supp_pls050_pls050supp.ppt]
